# Mechanistic Insights on Heme-to-Heme Transmembrane Electron Transfer Within NADPH Oxydases From Atomistic Simulations

**DOI:** 10.3389/fchem.2021.650651

**Published:** 2021-05-04

**Authors:** Xiaojing Wu, Jérôme Hénin, Laura Baciou, Marc Baaden, Fabien Cailliez, Aurélien de la Lande

**Affiliations:** ^1^CNRS, Université de Paris, UPR 9080, Laboratoire de Biochimie Théorique, Paris, France; ^2^Institut de Biologie Physico-Chimique-Fondation Edmond de Rotschild, PSL Research University, Paris, France; ^3^Institut de Chimie Physique, Université Paris Saclay, CNRS (UMR 8000), Orsay, France

**Keywords:** Marcus theory of electron transfer, reaction free energies, NADPH oxydase, molecular dynamics simulations (MD simulations), membrane protein, electron tunneling

## Abstract

NOX5 is a member of the NADPH oxidase family which is dedicated to the production of reactive oxygen species. The molecular mechanisms governing transmembrane electron transfer (ET) that permits to shuttle electrons over the biological membrane have remained elusive for a long time. Using computer simulations, we report conformational dynamics of NOX5 embedded within a realistic membrane environment. We assess the stability of the protein within the membrane and monitor the existence of cavities that could accommodate dioxygen molecules. We investigate the heme-to-heme electron transfer. We find a reaction free energy of a few tenths of eV (ca. −0.3 eV) and a reorganization free energy of around 1.1 eV (0.8 eV after including electrostatic induction corrections). The former indicates thermodynamically favorable ET, while the latter falls in the expected values for transmembrane inter-heme ET. We estimate the electronic coupling to fall in the range of the μeV. We identify electron tunneling pathways showing that not only the W378 residue is playing a central role, but also F348. Finally, we reveal the existence of two connected O_2−_binding pockets near the outer heme with fast exchange between the two sites on the nanosecond timescale. We show that when the terminal heme is reduced, O_2_ binds closer to it, affording a more efficient tunneling pathway than when the terminal heme is oxidized, thereby providing an efficient mechanism to catalyze superoxide production in the final step. Overall, our study reveals some key molecular mechanisms permitting reactive oxygen species production by NOX5 and paves the road for further investigation of ET processes in the wide family of NADPH oxidases by computer simulations.

## Introduction

NADPH oxidases (NOX) encompass a family of membrane enzymes dedicated to the production of cellular reactive oxygen species (ROS) in a regulated manner (Geiszt and Leto, 2004; Bedard and Krause, 2007). NOX enzymes have conserved structures common to all family members. In Humans, seven isoenzymes are expressed: NOX1 to NOX5, Duox1 and Duox2. This family is subdivided into two groups; one according to the ability of the NOX to form a heterodimer with p22^phox^ (NOX1-4), the other according to the presence of calcium-binding EF-hand type motifs in its sequence (NOX5 and Duox1/2). These enzymes are expressed in many tissues, including kidney, fibroblasts, osteoclasts, and thyroid, where they produce ROS, albeit generally at diverse levels, in response to stimuli such as growth factors, cytokines or calcium. They are involved in a great number of physiological functions, such as host defense, post-translational proteins processing, inter- and intra-cellular signaling, regulation of gene expression and cell differentiation. Aberrant levels of NADPH-oxidase derived ROS, either too low or too high, can disturb the balance of cellular homeostasis, ultimately resulting in pathological states. NOX are widely distributed in different kingdoms of life including animals (in particular mammals), plants and lower organisms and their expression is specific to each kingdom of life (Bedard et al., 2007; Sumimoto, 2008; Hajjar et al., 2017).

**Figure 1:**
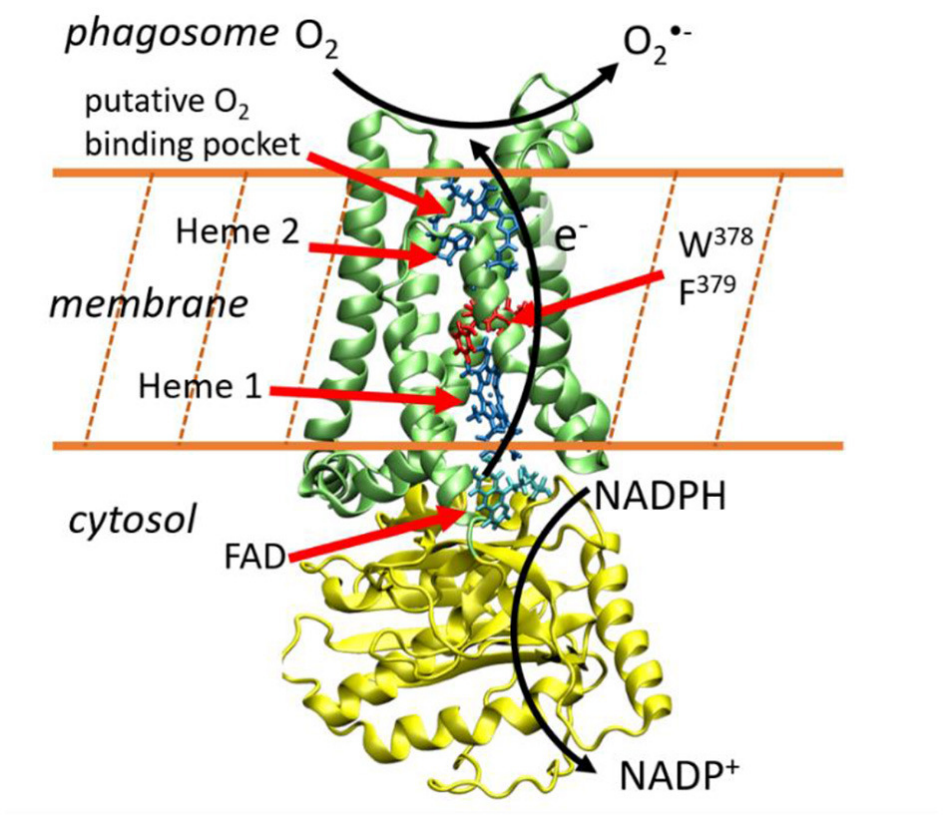
Electron transfer across the membrane catalyzes the production of superoxide anions. This image has been generated using the NOX5 model reported in Magnani et al. (2017). The yellow and green protein domains are the dehydrogenase and transmembrane domains respectively.

Whereas the members of the NADPH oxidase family differ in their tissue distribution, mode of activation and physiological functions, they all share the capacity to generate ROS. Among them, superoxide is produced by NOX1-3 and NOX5, and H_2_O_2_ is produced by NOX4 and Duox enzymes. NOX are flavohemoproteins and electron transporters consisting in a N-terminus region of six transmembrane helices that bind two non-identical heme groups and a cytosolic C-terminus region (dehydrogenase domain) where an NAD(P)H and a Flavin Adenosine Diphosphate (FAD)-binding site are localized. This transmembrane protein permits to shuttle electrons brought by NADPH molecules from the cytosol toward the internal part of the phagosome (Figure 1). Directional electron transfers are catalyzed by a series of redox molecules encapsulated within the protein matrices that serve as stepping stones for the electrons. On the cytosolic side, FAD is the primary electron acceptor and gets a hydride anion, *i.e*. two electrons and one proton, from NADPH. It is hypothesized that electrons are then shuttled one-by-one to the first heme (Heme1), then to the second heme (Heme2), before being finally transferred to a dioxygen molecule to form the superoxide anion. Redox potentials of hemes in NOX2, which is the most studied member of the NADPH oxidase family and is considered as the prototype of NOX family, have been measured (Cross et al., 1995). To the best of our knowledge, there have been no reports of kinetic measurements of these intrinsic ET steps which are presumably faster than other rate-limiting steps of the overall NOX machinery (*e.g.*, sub-units association or NADPH binding).

This sketchy description of transmembrane ET is supported by various biochemical data, notably by recent 3D structures. In 2017, Magnani et al. reported the first X-ray diffraction structures of the dehydrogenase and transmembrane domains of NOX5 from *Cylindrospermum stagnale*. This cyanobacteria NOX5 exhibits 40% sequence homology with human NOX5 (Magnani et al., 2017). Very recently, cryo-EM structures of mouse Duox1–DuoxA1 have been reported (Sun, 2020). NOX5 and Duox catalyze superoxide and hydrogen peroxide production, respectively. Their 3D-structures are well conserved and present two well-aligned hemes in the transmembrane domain (Figure 1). A phenylalanine residue (379 in NOX5 and 1097 in DUOX1-DUOXA1) is located midway between the two hemes suggesting this residue could play an important role to sustain inter-heme electron tunneling. These ET path schemes in NOX enzymes are valuable hypotheses but should be taken with caution as reasoning on single structures to predict tunneling pathways, ignoring structural fluctuations, has often proven to be misleading (Prytkova et al., 2007; de la Lande et al., 2010, 2013; Beratan et al., 2015). Putative binding pockets for O_2_ molecules have been identified which could serve to maintain molecular oxygen close to Heme2, thus facilitating its reduction (Magnani et al., 2017; Sun, 2020).

Globally, we start to accumulate structural insights on NOX enzymes. However, little is known about the thermodynamics and kinetics of the succession of electron transfer steps within NOX. In this article, we report the first microscopic simulations of NOX5 within a realistic lipid membrane. Our aim is twofold. First, we aim at building a realistic model of NOX5 at the atomic level. We report molecular dynamics simulations of several hundreds of nanoseconds to assess its stability in a fully hydrated lipid bilayer environment. Second, we report a computational study of the inter-heme electron transfer step including evaluations of the ET free energy, of reorganization free energies and of tunneling pathways. We further reveal that O_2_ moves back-and-forth between two binding pockets situated near Heme2. When Heme2 is reduced, O_2_ dominantly populates the pocket which is the closest to the heme. The electron tunneling pathways afforded by this pocket are one order of magnitude stronger than those emerging when O_2_ is present in the more remote pocket, hence providing a way to catalyze the final electron transfer to produce superoxide. Data and scripts to produce key figures and table of this article are available on the Zenodo data base (doi: 10.5281/zenodo.4424142).

## Building of an Atomistic Model for NOX5

Our aim is to investigate inter-heme electron transfer. To this end, our approach is to develop an atomistic model of NOX5 inserted within a lipid bilayer based on the recently reported structures of helical transmembrane (TM) and C-terminal cytosolic dehydrogenase (DH) domains from *Cylindrospermum stagnale* NOX5. The initial coordinates of the TM and DH domains were taken from the Protein Data Bank structures with codes 5O0T (2.0 Å resolution) and 5O0X (2.2 Å resolution), respectively. We start by detailing the protocol we have followed to set-up the system and to carry out molecular dynamics simulations. In the following section we will focus on electron transfer modeling.

### Molecular Dynamics Simulation of NOX Proteins

#### Preparation of the Simulation System

**Figure 2:**
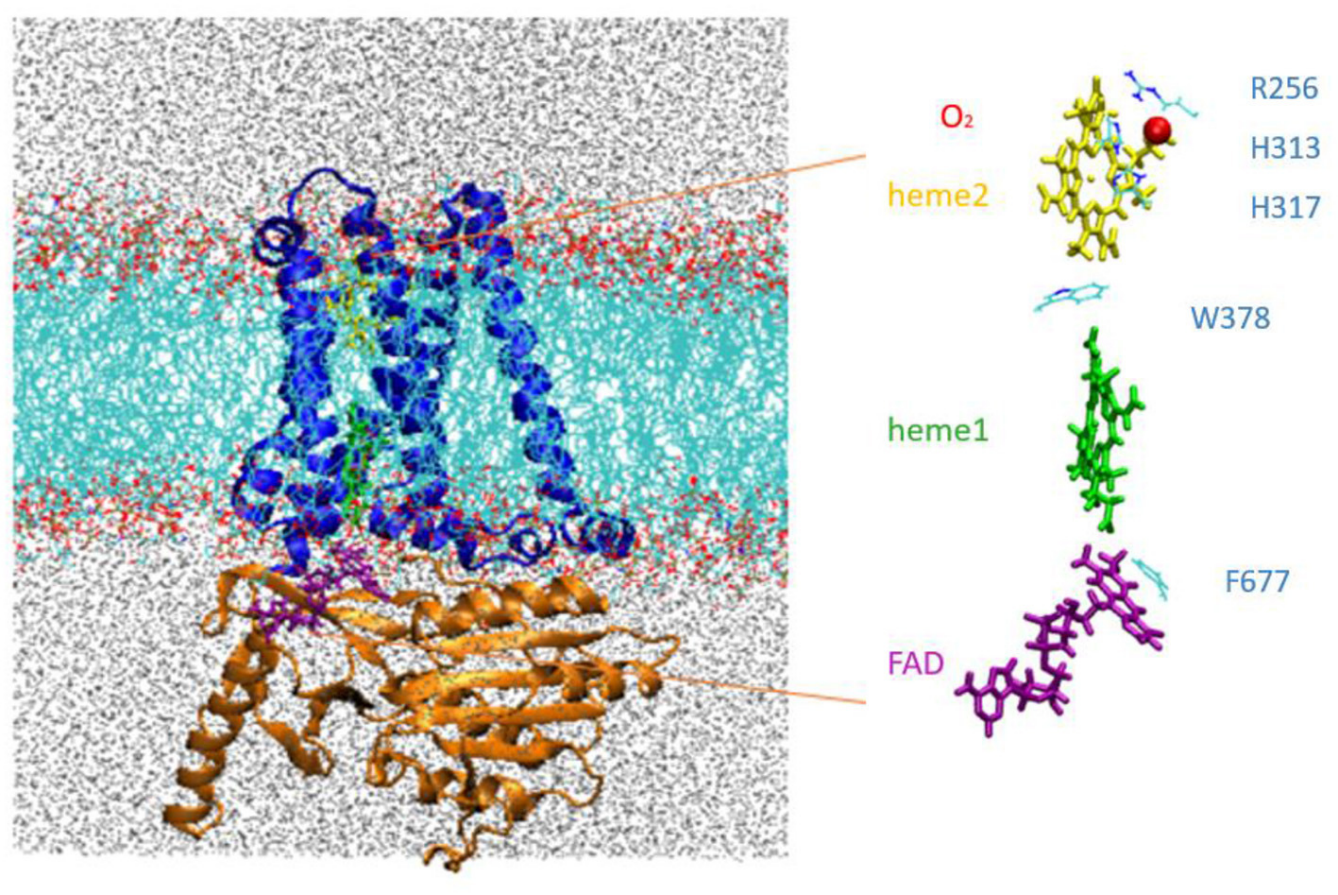
Left, NOX5 catalytic core investigated in this study, composed of the transmembrane domain (in blue) and dehydrogenase domain (in orange) embedded in a lipid bilayer (in light blue and red). Water molecules are shown in gray. Right, insert shows the cofactor FAD (purple), Heme1 (green), Heme2 (yellow) and O2 (red).

The MD simulations were carried out for an extended system comprising the NOX protein catalytic core embedded in a model phospholipid membrane and solvated with an ionic aqueous solution as shown in Figure 2. Magnani et al. (2017) identified a putative cavity to bind a dioxygen molecule in the vicinity of Heme2 (see the red bead on Figure 2, right). In our simulations this cavity is filled either by a dioxygen molecule or by a water molecule (see below).

### Construction of a Full-Length NOX5 Model

In the experiments that achieved crystallization of the DH domain, a short amino acid sequence PWLELAAA was added after the C-terminal Phe693 residue to enhance thermal stability and FAD retention (Magnani et al., 2017). We removed this artificial additional sequence in our model. On the other hand, 27 amino acid residues were missing in the N-terminal calcium-binding EF-hand domain (GAQQKSDMKSSTLFVAMDLMHQETKVD), for which no homology modeling template was available. We used the PSIPRED server (Buchan and Jones, 2019) to predict the secondary structure of this segment. The central part of this sequence is predicted to form an alpha helix. MODELLER (Šali and Blundell, 1993) was used to add this missing part, however the resulting models did not form an alpha-helix. This segment is only present in the NOX5 branch of the family, and is replaced by e.g., an 11-residue stretch in a NOX4. So, we constructed a NOX4-like variant, by deleting SSTLFVAMDLMHQET and only keeping GAQQKSDMKVD (Resid 605–615). This shorter sequence was modeled *ab initio* with MODELLER. 20 models were built and assessed based on their DOPE (Discrete Optimized Protein Energy) score (Shen and Sali, 2006). Differences between the 20 models were small: the RMSD difference between the model with best score and worst is 0.19 Å. We considered that 20 models are enough to build this missing part and we chose the model with the best score. We do not expect this assumption to influence the study of the ET process between two hemes, since the distance between the reconstructed loop and Heme1 is 35 Å.

In ref. Magnani et al. (2017), the X-ray structures of the two DH and TM domains were obtained separately, and docked together with some user-defined restraints using the Haddock server (Dominguez et al., 2003; van Zundert et al., 2016). We have followed the same docking strategies. Specifically, Asn288 (B-loop of TM), Lys361 (D-loop of TM), Thr520 (B-loop binding region of DH), and Phe677 (flavin-interacting C terminus of DH) were defined as “active” residues (preferentially placed at the interface by Haddock). Moreover, we constrained the edge-to-edge distance between FAD and Heme1 to be within 2.5 Å. We also constrained Phe677 to be close to the isoalloxazine ring of FAD with a face-to-face π-stacking interaction (Figure 2). The distance between Lys412 (C terminus of TM) and Glu413 (N terminus of DH) was constrained to 2 Å. The best docked complex (Haddock score of −2.5) was then fused by creating a covalent bond between Lys412 and Glu413. The geometry of the model was further validated by the Qmean server (Benkert et al., 2011) for model quality estimation. Our model obtained a QMEAN Z-score of −1.97. We can consider this score as satisfactory since the Duox1 cryo-EM structure has a slightly worse Z-score of −2.65.

The interface between the transmembrane and dehydrogenase domains was the main unknown when building our full-length model. Recent cryo-EM structures of the homologous Duox1 protein contain both domains, and offer a basis for comparison (Sun, 2020). We find that when aligning the transmembrane domain, our model of the dehydrogenase domain is rotated by 60° with respect to the equivalent domain in the 7D3E structure of the human DUOX1-DUOXA1 complex (Supplementary Figure 1). During the simulations, the relative orientation of the two domains fluctuates by up to 11° from the initial model.

### Construction of the Extended System

The docking structure was used as initial coordinates of the catalytic core. All amino acid residues were assigned a standard protonation state at pH 7. All histidine residues were singly protonated at the Nδ sites based on the inspection of inter-residue interactions. The importance of the lipid metabolism was evidenced in NOX activities, although the regulatory role of the lipid membrane properties remains unclear. Membrane properties (charges, thickness, composition, lipid raft formation) have been shown to affect the NOX2 enzyme functioning (Shao et al., 2003; Souabni et al., 2014, 2017). Moreover, the phospholipid composition changes during NOX2 activation (Magalhaes and Glogauer, 2010; Bréchard et al., 2012; Joly et al., 2020). We made the choice of building a membrane with 1-Palmitoyl-2-oleoyl-sn-glycero-3-(phospho-rac-(1-glycerol)) (POPG) and 1-palmitoyl-2-oleoyl-sn-glycero-3-phosphoethanolamine (POPE) in 4 to 1 proportions with CHARMMGUI (Jo et al., 2008; Brooks et al., 2009; Lee et al., 2016). Note that this membrane composition is one among many possibilities that could have been considered. The OPM (orientations of proteins in membranes) orientation of the TM domain has been used as starting point for membrane insertion. The protein membrane system was solvated with 38,341 water molecules in a hexagonal prism unit cell (a,b,c = 135Å,117Å,112 Å, alpha, beta, gamma = 90, 90, 120°). The system was neutralized electrically by adding 451 Na+ and 98 Cl− ions to obtain a 0.15 M NaCl solution.

### Force Field Parameters

Standard protein residues and lipids were modeled using the CHARMM36 force field (Lee et al., 2016). We used the TIP3P water model (MacKerell et al., 1998). Force field parameters for the heme cofactor in ferrous and ferric states and for FADH• have been developed by our groups and can be found in Supplementary Materials. In this study, we consider two distinct redox states associated to the inter-hemes electron transfer (see next section): Heme1-Fe(II)/Heme2-Fe(III) and Heme1-Fe(III)/Heme2-Fe(II). In some simulations, a single dioxygen molecule was placed in the cavity shaped by the propionate groups of Heme2 and by residues Arg256, His313 and His317 as shown in Figure 2. This cavity has been previously proposed to host dioxygen prior to its reduction. Following the methodology proposed in Ref. Javanainen et al. (2017, 2020), we placed a charged dummy atom holding a 0.452 electric charge in between the oxygen nuclei, each holding a −0.226 electric charge in order to reproduce the quadrupole moment of the dioxygen molecule. A restraint energy term was added to the total potential energy with the Colvar module (Fiorin et al., 2013) of NAMD (Phillips et al., 2005, 2020) to maintain the dioxygen molecule within the pocket during the MD simulations (details are given later in the text).

### Simulation Protocol

Initial equilibration and production phase were carried out using the NAMD program (Phillips et al., 2020). We carried out MD simulations for two different systems. One system includes the presence of O_2_ in the aforementioned cavity, while the other is without dioxygen. We conducted in each case MD simulations for the system in the initial and final redox states, represented by different partial charge distributions on heme and iron moieties. The protein membrane system was equilibrated with a standard procedure as suggested from CHARMM-GUI (Jo et al., 2008; Lee et al., 2016). The equilibration phase has been carried out in the isothermal–isobaric ensemble (NPT) under periodic boundary conditions with six cycles of 25, 25, 25, 200, 200, 200 ps by gradually releasing harmonic restraints around the initial positions of protein backbone and membrane lipid atoms at 310 K and 1 bar. For protein, the harmonic restraints force constants of each cycle were set to 10.0, 5.0, 2.5, 1.5, 1.0, and 0.5 kcal/mol/Å^2^, while membrane lipid restraints were applied with force constants of 5.0, 5.0, 2.0, 1.0, 0.2, and 0 kcal kcal/mol/Å^2^. The integration time step was set to 1 fs for the first three cycles and then increased to 2 fs for the last three cycles. All bonds involving H are fixed in length. To maintain planarity of the membrane, the center of mass of the lipid head groups in the upper and lower layers had their positions restrained in the z direction to +19 and −19 Å relative to the center of mass of the bilayer by application of a constraining potential with a force constant of 5.0, 5.0, 2.0, 1.0, 0.2, and 0 kcal/mol/Å^2^. The particle-mesh Ewald (PME) method was used for the calculation of electrostatic interactions. Non-bonding interactions were treated using a cutoff of 12.0 Å. Pressure was controlled by the Nose-Hoover Langevin piston method (Phillips et al., 2005) while the temperature was controlled by Langevin dynamics.

Production runs in the canonical ensemble (NVT) were carried out for 300 ns for each redox state. Geometries were saved every 2 ps to sample the vertical energy gap. We also conducted two 100 ns MD simulations for each state with the same equilibrated structure but different initial velocities to assess the variability of the computed ET parameters with respect to the initial conditions of the simulation. Altogether, we generated a set of 12 MD simulations, 6 for each state (with and without oxygen), with 3 replicas of 300, 100, and 100 ns for each state, respectively, and two charge distributions each representing the initial and final redox states.

### Analysis of Protein Stability

The protein backbone root-mean-square deviation (RMSD) time series calculated during MD simulations are shown in Supplementary Figure 2. The docking structure is taken as reference for the RMSD calculation. Panels (a) to (c) correspond to the MD with oxygen present, while panels (d) to (f) are without O_2_. Black (resp. red) curves correspond to dynamics performed in the initial (resp. final) redox states. For all simulations, the RMSD stays under around 4 Å, assessing a slight distortion with respect to the initial structural model. Only in the 300 ns-long MD simulation without O_2_ in the initial redox state a small drift toward higher values of RMSD is seen at the end of the simulation.

### Analysis of NOX5 in the Membrane

We have assessed the stability of the insertion of the transmembrane domain of the protein in the lipid bilayer by computing the membrane thickness and the relative position of the protein in the bilayer throughout the simulations. The membrane thickness is estimated by the distance between the center-of-mass of upper and lower lipids. It is stable during all the MD simulations with a fluctuation of less than 2 Å (Supplementary Figure 3). We use the distance between the center of mass of the membrane and the center of mass of the transmembrane part of the protein to measure the relative position of the protein inside the bilayer. Its evolution in all the MD simulations is shown in Supplementary Figure 4. It is found to be stable with fluctuations less than 6 Å and no apparent drift on the hundreds of ns timescale.

### Heme-to-heme Electron Transfer

Having assessed the stability of the *in-silico* model of NOX5 inserted in a lipid membrane, we focus in this section on the heme-to-heme electron transfer. We follow the framework of the Marcus Theory of electron transfer to evaluate the free energy of the reaction (Δ*A*°) and the reorganization energy (λ) (Marcus and Sutin, 1985). Warshel et al. showed that both quantities can be obtained from microscopic simulations under the Linear Response Approximation as (King and Warshel, 1990):

(1) $$\begin{array}{r} \Delta A^{0}=\frac{1}{2}\left({\langle \Delta E\rangle }_{i}{+\langle \Delta E\rangle }_{f}\right) \end{array}$$

(2) $$\begin{array}{r} \lambda^{St}=\frac{1}{2}\left({\langle \Delta E\rangle }_{i}{-\langle \Delta E\rangle }_{f}\right) \end{array}$$

where Δ*E* is the diabatic energy gap, that is the difference in potential energy of the system in the initial and final redox state (Δ*E* = *E*_*f*_−*E*_*i*_), or alternatively said when the transferred electron sits on Heme1 or Heme2, respectively. Note that Δ*E* is calculated for a given set of nuclear coordinates and is therefore referred to as a vertical energy gap. 〈…〉_*x*_ refers to an average performed over a canonical ensemble of configurations of the system in redox state *x* (*i* or *f*). Δ*E* can be decomposed into a contribution coming from the heme cofactors (inner-sphere contribution), from the environment (outer-sphere contribution) and from a coupling term between them. The latter refers to the mutual polarization (*E*^*mp*^) that differs for the two redox states: Δ*E* = Δ*E*^*is*^+Δ*E*^*os*^+Δ*E*^*mp*^. Neglecting Δ*E*^*mp*^, a separation of the total free energy of the reaction as Δ*A* = Δ*A*^*is*^+Δ*A*^*os*^ is obtained. This approximation has been tested in other heme proteins and turned out to be reasonable (Blumberger, 2008). We adopt it here, too. Furthermore, as ET is taking place between two chemically identical hemes, Δ*A*^*is*^ = 0, therefore Δ*A*≈Δ*A*^*os*^.

The reorganization energy calculated with Equation 2 is referred to as the Stokes reorganization energy, hence the upper script *St*. The fluctuation of the energy gap can also be used to define the reorganization energy:

(3) $$\begin{array}{r} \lambda_{x}^{var}=\frac{var\left(\Delta E_{x}\right)}{2k_{B}T} \end{array}$$

In Equation 3, Δ*E*_*x*_ is the energy gap computed over a trajectory performed in the state *x* (*i* or *f*). From MD simulations performed on each electronic state involved in the ET, one obtains two different values: $\lambda_{i}^{var}$ and $\lambda_{f}^{var}$. If the LRA and the ergodic principle apply, the three definitions of the reorganization energy coincide: $\lambda^{St}=\lambda_{i}^{var}=\lambda_{f}^{var}$. A simple measure of non-ergodicity can be obtained as:

(4) $$\begin{array}{r} \chi_{G}=\frac{\lambda_{i}^{var}+\lambda_{f}^{var}}{2\lambda^{St}} \end{array}$$

Values of χ_*G*_ close to 1 correspond to ergodic systems.

Neglecting Δ*E*^*mp*^ as above, reorganization energy can be decomposed into two contributions: λ = λ^*is*^+λ^*os*^. λ^*os*^ has been evaluated by Eqs. 2 and 3 using force field-based interaction energies computed between the two hemes and the environment. λ^*is*^ has been evaluated by Density Functional Theory calculation (see Supplementary Material for details). The geometries of the heme cofactors including the apical and axial histidine ligands (modeled as methyl-imidazoles) have been optimized and λ^*is*^ has been calculated as:

(5) $$\begin{array}{r} \lambda^{is}=2.\left(\frac{E_{gR}^{O}-E_{gR}^{R}+E_{gO}^{R}-E_{gO}^{O}}{2}\right) \end{array}$$

**Figure 3:**
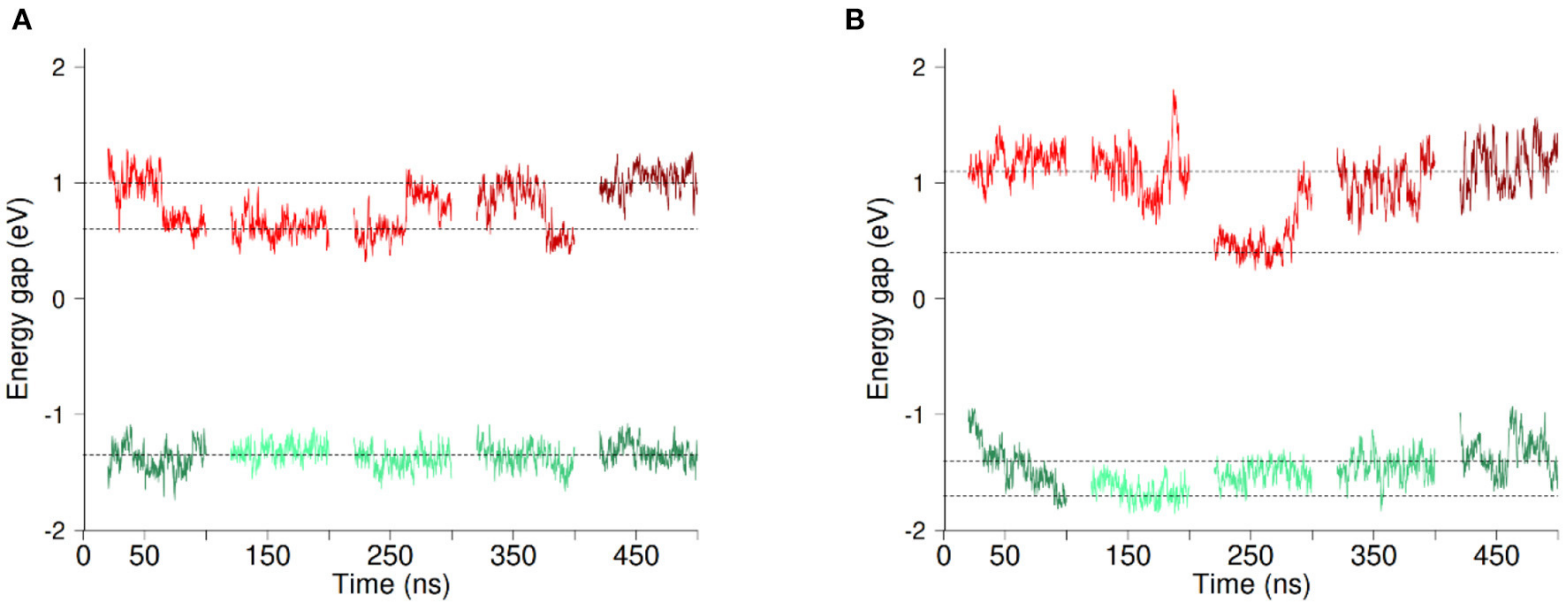
Evolution of the vertical energy gap Δ*E* (*E*_*f*_−*E*_*i*_) along MD simulations with O_2_ **(A)** and without O_2_ **(B)**. Each line section represents 80ns of MD simulation. The first three sections correspond to parts of the 300ns-long simulation (Set 1–3) while the two last sections come from the two independent 100ns-long replica simulations (Set 4 and 5). Energy gaps computed based on simulations in the initial (resp. final) redox state are shown in red (resp.green). Data shown correspond to running averages of Δ*E* for clarity. Horizontal lines are guides to the eye.

**Figure 4:**
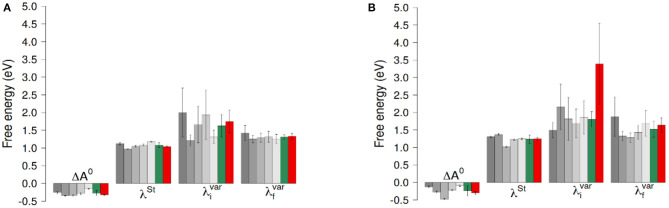
ET reaction free energies and outer-sphere reorganization energies of the ET between hemes with O_2_ **(A)** and without O_2_ **(B)**. The gray bars represent values obtained for the five 100ns-long data sets. The green bars are the results of a block average using the data of these five data sets. The red bars correspond to values obtained using the full 300ns-long trajectory. Error bars correspond to twice the uncertainty.

$E_{gX}^{X}$ stands for the energy of the heme in the redox state *X*(*O*∨*R*) in the equilibrium geometry *gX*. We obtained a value of 0.10 eV for λ^*is*^. For each system (with or without O_2_) we have computed ET thermodynamic parameters using the full 300 ns-long MD simulations, discarding the first 20 ns. In order to assess the variability of ET parameters with respect to the sampling, we have split the 300 ns-long simulations into three segments of 100 ns. Adding the two small 100 ns-long simulations, we have access to 5 sets of data that we consider to be independent, hereafter referred to as Set1 to Set 5. From these datasets, we have computed ET thermodynamic parameters, discarding each time the first 20ns, considered as an equilibration time. A block analysis over the 5 obtained sets of values has finally been performed. The results obtained for Heme1 to Heme2 ET are summarized in Figures 3, 4 (all the individual numerical values are given in Supplementary Table 1).

Figure 3 presents the evolution of the energy gaps Δ*E* in the five data sets Set1 to Set5 with O_2_ (panel a) or without O_2_ (panel b), computed in the initial (in red) and final (in green) redox states. We begin by describing the results in presence of dioxygen. The data clearly shows that fluctuations of the energy gap are greater in the initial redox state (in red). What is more, a clear bimodality appears for the energy gaps in this redox state, with Δ*E* oscillating around two values, which are indicated with horizontal dotted lines in Figure 3. This oscillation seems to take place at the scale of tens of ns. No clear-cut localized change in structure of the protein and/or its environment could be found to account for this bimodality. That said, we decomposed Δ*E* into different contributions, namely TM domain, DH domain, environment (water, membrane and counter ions) and cofactors (FAD, hemes, O_2_). The result of this decomposition is shown in Supplementary Figures 5, 6 for simulations in presence and absence of O_2_ respectively. For the initial redox state we recover a bimodality when considering the contributions from the TM domain and from the environment. Longer simulations could help to decipher the origin of the bimodality of Δ*E*. It could come from slow concerted motion involving the TM and the lipids composing the membrane and its solvation layer. On the other hand, the other components (FAD, Hemes, O_2_, DH domain) don't exhibit any bimodality in the energy gap distributions. In addition, these results are independent on the presence or not of dioxygen in the cavity.

ET reaction free energies obtained with Equation 1 are negative whatever data set is used to compute them, with values ranging roughly from −0.5 to −0.1 eV (see Figure 4 and Supplementary Table 1), indicating that the ET from Heme1 to Heme2 is favorable. The values obtained from the full 300 ns-long MD simulations or from the five 100 ns-long datasets are consistent (green and red bars in Figure 4). Because of the larger fluctuations in Δ*E*, values of Δ*A*^0^ obtained without O_2_ are more dispersed (see e.g., Figure 4). However, the average values obtained for the two systems are similar within the uncertainties of the calculations (−0.28 eV and −0.24 eV respectively with and without O_2_). Outer-sphere reorganization energies are presented in Figure 4, calculated with Equation 2 (λ^*St*^) or Equation 3 ($\lambda_{i}^{var}$ and $\lambda_{f}^{var}$). λ^*St*^values lie between 1 and 1.4 eV, with averages of 1.04 and 1.24 eV for λ^*St*^ respectively with and without O_2_. Note that these reorganization energies are computed with a non-polarizable forcefield. Accounting for electrostatic induction is expected to decrease the values of the reorganization energies by 30% approximately (Blumberger, 2008). Once again one observes greater fluctuations in the values when O_2_ is not bound to Heme2. This is especially the case when one considers $\lambda_{x}^{var}$ values computed over the 300 ns-long MD simulations in the initial electronic state. This observation should be taken with caution as the duration of the simulation is likely not to be long enough with respect to the slow degree of freedom that is responsible for the bimodality in Δ*E*. Whichever way they are computed, reorganization energies are greater in absence of dioxygen (see Supplementary Table 1). This dependency may arise because the presence of O_2_ “rigidifies” the environment around Heme2, lowering the structural differences between both electronic states, noting that the uncertainties of the mean values are large, respectively 0.07 and 0.24 eV. Further investigations with longer simulations or more replicas should help to ascertain these conclusions more strongly. Values of $\lambda_{x}^{var}$ are found to be greater than λ^*St*^ for every dataset, leading to χ_*G*_ values between 1.1 and 2.0, most of the time lying between 1.3 and 1.5. For simulations without O_2_, the long 300 ns MD simulations gives rise to a much larger $\lambda_{i}^{var}$(ca. 3.5 eV, red bar in Figure 4, right). This high value, associated to a large statistical uncertainly (1.17 eV, Supplementary Table 1) results from the bimodality in the energy gap distribution which is apparent in Figure 3. Together with the low values obtained for λ^*os*^, this finding reveals that the structural reorganization around the two hemes is somewhat moderate, since much larger χ_*G*_ values have been reported (Matyushov, 2015).

We have decomposed Δ*A*^0^ and λ^*St*^ into contributions arising from the molecular components of the system, namely the TM and DH domains, FAD, the Hemes, the counter-ions, the membrane and the solvent (Supplementary Table 2). It turns out that the negative Δ*A*^0^ results from a balance between positive and negative contributions. With the current membrane composition (POPG/POPE in 4:1 proportion), the TM domain and membrane largely disfavor inter-heme ET (1.52 ± 0.18 and 0.60 ± 0.14 eV, respectively, considering the average over the five sets of trajectories), while water and counter-ions provide a net driving force for ET (−0.27 ± 0.12 and −1.42 ± 0.68, respectively). We can thus extrapolate that a change of membrane composition, especially if it incorporates more of less charged lipids would directly impact inter-heme ET. Interestingly, the DH domain has little influence on Δ*A*^0^ and marginally reorganizes upon electron transfer. As in our model the DH domain might have a non-physiological orientation with respect to the TM domain in view of the recent cryo-EM structure of DUOX1 (see above, and Supplementary Figure 1), this result is reassuring regarding our estimates of inter-heme ET thermodynamics, *i.e*., the precise orientation of the DH domain marginally determines these parameters, likely because the DH is far away from the inter-heme region.

### Electron Tunneling Pathways

We have carried out tunneling pathway analyses along MD trajectories with the pathway model of Beratan and co-workers. This model is built on the assumption that the electron tunnels from the electron donor to the acceptor along a pathway that is defined as a succession of covalent or hydrogen bonds and of through space contacts (Beratan et al., 1987). The decay of the electronic coupling associated with a given pathway is expressed as the product of multiplication of a constant contact coupling ($H_{if}^{contact}$) by a factor (ε_*tot*_) that reflects the attenuation of the coupling caused by the presence of intervening medium between the donor and the acceptor. ε_*tot*_ depends on the number of covalent or hydrogen bonds (denoted *N*_c_ and *N*_hb_, respectively) and of through-space jumps (*N*_ts_) composing the electron transfer pathway. This factor is calculated according to a set of mathematical expressions empirically calibrated by the authors of the model (Onuchic and Beratan, 1990; Beratan et al., 1991).

(6) $$\begin{array}{r} H_{if}=H_{if}^{contact}\varepsilon_{tot} \end{array}$$

(7) $$\begin{array}{r} \varepsilon_{tot}=\underset{N_{c}}{\prod }\varepsilon_{c}\times \underset{N_{hb}}{\prod }\varepsilon_{hb}\times \underset{N_{ts}}{\prod }\varepsilon_{ts} \end{array}$$

(8) $$\begin{array}{r} \varepsilon_{c}=0.6 \end{array}$$

(9) $$\begin{array}{r} \varepsilon_{hb}=0.36\times exp\left[-\beta_{S}\left(R_{H}-2.8\right)\right] \end{array}$$

(10) $$\begin{array}{r} \varepsilon_{ts}=0.6\times exp\left[-\beta_{S}\left(R_{S}-1.4\right)\right] \end{array}$$

**Figure 5:**
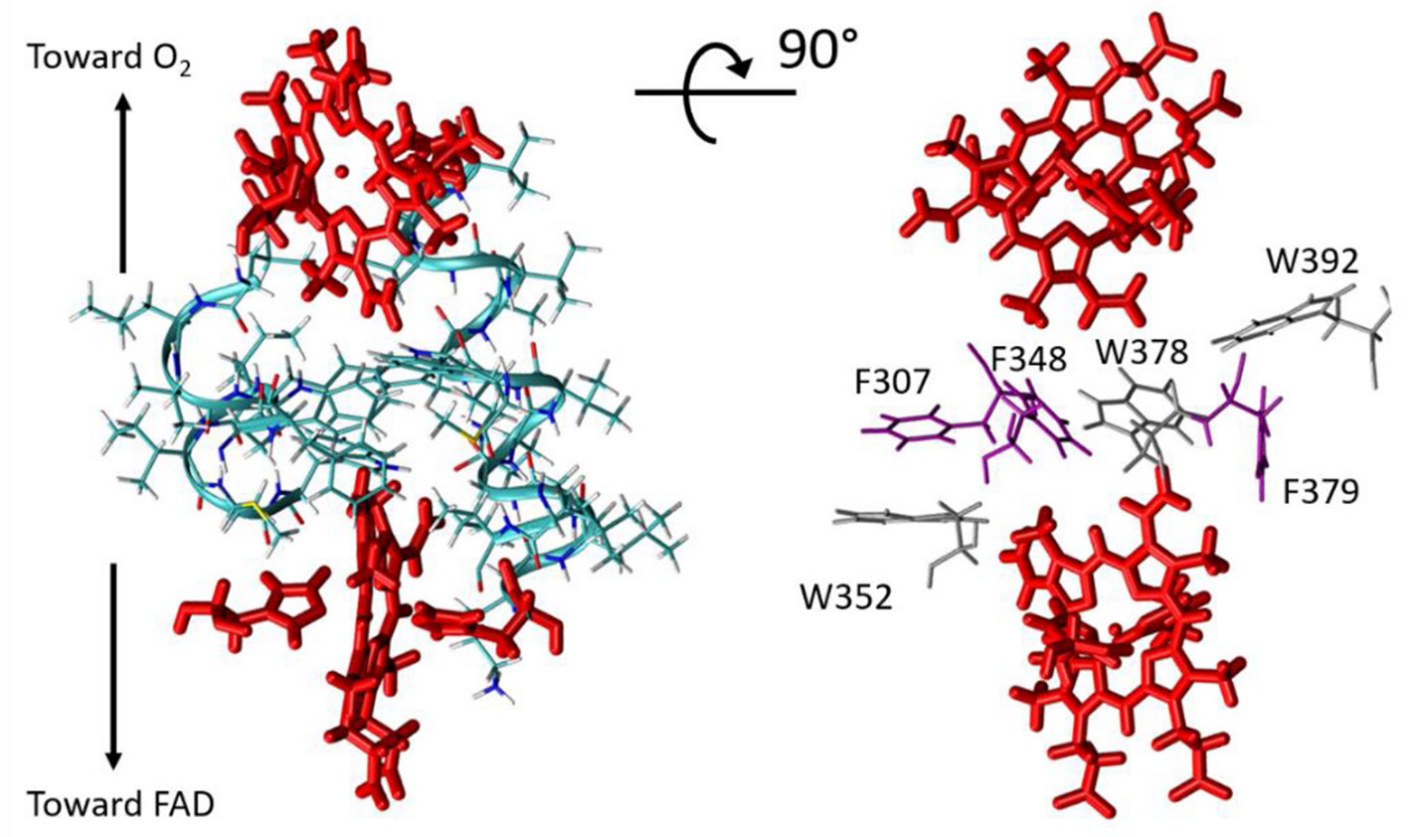
Left, restricted molecular system considered in ET pathway analyses. The hemes are shown in red. Right, for clarity only the closest residues from the Donor-Acceptor axis are represented (tryptophan residues in gray and phenylalanine residues in violet).

*R*_*S*_ is the atom–atom distance for through-space jumps, *R*_*H*_ is the hydrogen bond length (taking the inter-heavy atom distance) and β_*S*_ is a characteristic decay factor. The latter was originally parametrized in the 1990's to a value of 1.7Å^−1^, but later investigations suggested that a value of 1.1Å^−1^ enables better agreement with quantum chemistry calculations (Prytkova et al., 2005). This is the value we have used here. Covalent bonds within the porphyrin aromatic rings were made fully conductive (*i.e*., setting ε_*c*_ = 1 between two such atoms) in our analysis, so that the PM results do not depend on the particular choice of the donor/acceptor atom within the two hemes. The best pathway was identified for each analyzed structure using the Dijkstra algorithm (Dijkstra, 1959) with an in-house program (de la Lande et al., 2010; El Hammi et al., 2012). Our program (i) seeks for the pathway producing the most efficient electronic coupling with the Dijkstra algorithm, (ii) decomposes the total pathway over covalent, through-hydrogen-bond and through-space interactions (iii) indicates which amino-acid residues or other molecules are involved in tunneling pathways, (iv) provides adequate files for visual representation of the pathways with the VMD program. Structures were extracted from MD simulation trajectories every 20 ps. To make the pathway search tractable, the structure was preliminarily pruned to include only the atoms susceptible to take part in the tunneling process, that is residues not lying too far from the direct inter-heme axis. The pruned sub-system comprises the two hemes and their axial ligands (His299, His313, His372, His385) as well as resides Leu268, Ile269, Lys300, Leu301, Val302, Gly303, Gln304, Val305, Met306, Phe307, Ala308, Leu309, Ala310, Ile311, Val312, Leu345, Leu346, Val347, Phe348, Ile349, Ile350, Met351, Trp352, Trp378, Phe379, Trp392 (Figure 5). As seen on the picture, the hemes are separated by a layer of five aromatic amino acids, among which Trp378, as highlighted in Magnani et al. (2017) but also Phe307, Phe348, Trp352, Phe379, and Trp392.

**Figure 6:**
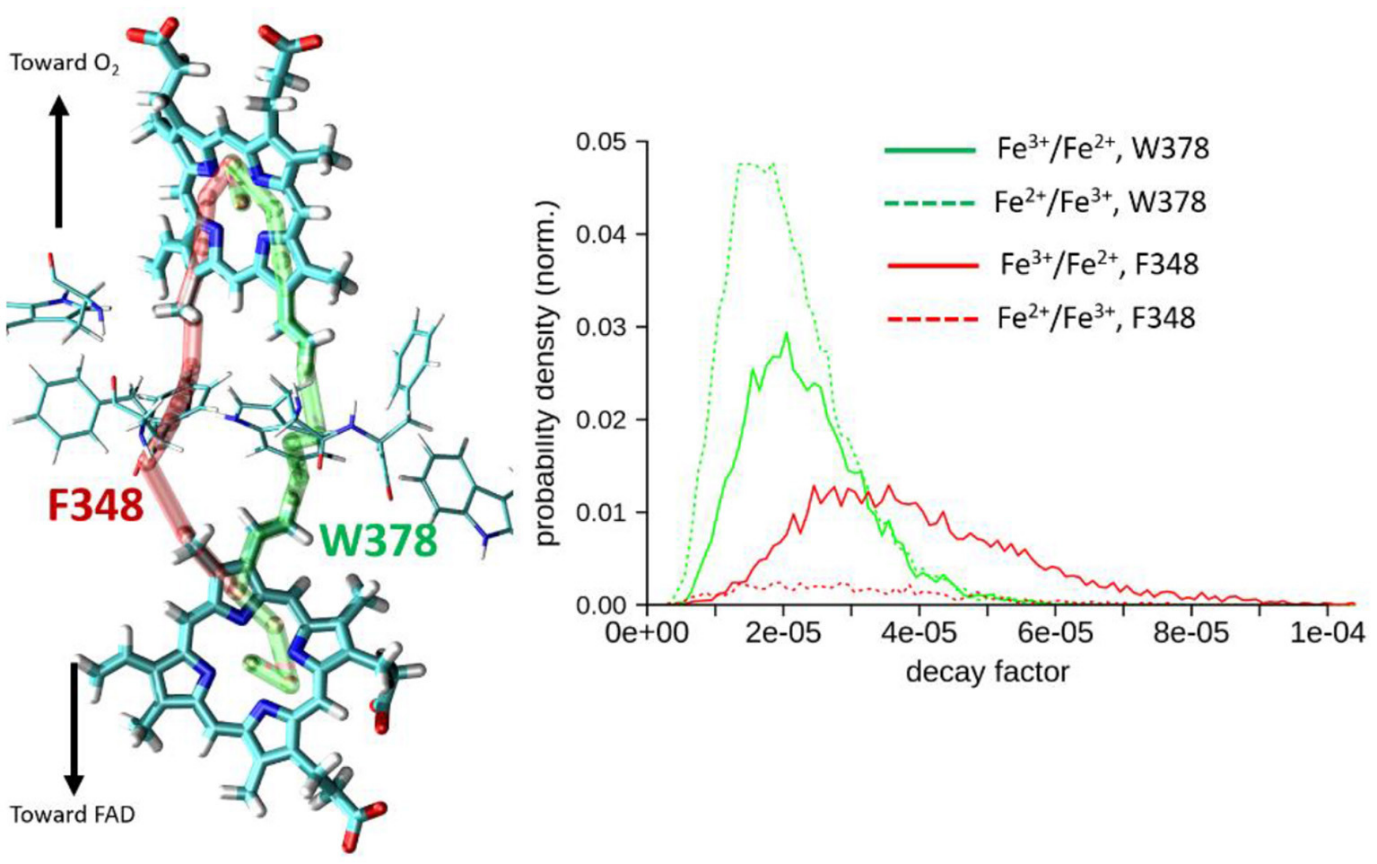
Left, illustration of the two types of pathways encountered for heme-to-heme ET in NOX5 running either through Trp378 (in green) or through Phe348 (in red). Right, distributions of tunneling decay factors for MD simulations in the Fe^2+^/Fe^3+^ and Fe^3+^/Fe^2+^ redox states (in dashed and plane lines respectively), deconvoluted by pathway types.

Results are collected in Table 1 and on Figure 6. Two types of pathways are identified over the duration of the MD simulations. The first one is running through Trp378, while the other is running through Phe348 as illustrated by the green and red tubes in Figure 6. They connect the hemes by the edge of the porphyrin plane. Our simulations thus confirm the expectation that Trp378 plays a key role in mediating inter-heme tunneling, but also reveal the occurrence of pathways running through Phe348. As seen on the distributions depicted in Figure 6, the tunneling pathways running through Phe348 are in general slightly more efficient than those running through Trp378. The respective distributions among the two kinds of pathways seem to depend on the redox state but it is difficult to find a rational in terms of structure of the intervening medium, and both kinds of pathways give rise to rather similar decay factors. Overall, the average decay factor equals to 2.11 10^−5^ with a standard deviation of 9.98 10^−6^ when analyzing MD trajectories of the Fe^2+^/Fe^3+^ redox state, and to 3.03 10^−5^ and 1.52 10^−5^ for the Fe^3+^/Fe^2+^ redox state.

**Table 1:** Semi-empirical pathway analyses of tunneling matrix elements mediating inter-heme electron transfer. $\langle H_{if}^{PM}\rangle$ are given in eV^2^.

**Path**	**(%)**	**〈ε_tot_〉**	**σ^2^**	**$\langle {\text{H}}_{\text{i}\text{f}}^{\text{P}\text{M}}\rangle$**	**$\langle {\varepsilon_{\text{t}o\text{t}}}^{2}\rangle$**	**${\text{R}}_{\text{c}o\text{h}}^{\text{c}\text{l}}$**
				**eV**		
**Fe**^**2+**^**/Fe**^**3+**^
Trp378	92	2.04 10^−5^	7.44 10^−11^	–	–	–
Phe348	7	3.06 10^−5^	2.58 10^−10^	–	–	–
Total	100	2.12 10^−5^	9.62 10^−11^	3.75 10^−6^	5.43 10^−10^	0.82
**Fe**^**3+**^**/Fe**^**2+**^
Trp378	56	2.32 10^−5^	7.83 10^−11^	–	–	–
Phe348	44	3.93 10^−5^	2.75 10^−10^	–	–	–
Total	100	3.04 10^−5^	2.30 10^−10^	5.40 10^−6^	1.15 10^−9^	0.80

As $\langle {\varepsilon_{tot}}^{2}\rangle ={\langle \varepsilon_{tot}\rangle }^{2}+\sigma^{2}$ with $\sigma^{2}=\langle {\left(\varepsilon_{tot}-\langle \varepsilon_{tot}\rangle \right)}^{2}\rangle$, the ratio $R_{coh}^{cl}$ defined as $R_{coh}^{cl}={\langle \varepsilon_{tot}\rangle }^{2}/\langle {\varepsilon_{tot}}^{2}\rangle$ permits to assess if tunneling is governed by fluctuations of the electronic coupling or by its average (Balabin and Onuchic, 2000; Balabin et al., 2012). If $R_{coh}^{cl}$ approaches unity it means that ${\langle \varepsilon_{tot}\rangle }^{2}\gg \sigma^{2}$, tunneling is governed by the average value. On the other hand, if $R_{coh}^{cl}$ approaches 0.5, it means that ${\langle \varepsilon_{tot}\rangle }^{2}\ll \sigma^{2}$, *i.e*. tunneling is likely to be dominated by the fluctuations. We obtain values larger than 0.8 suggesting tunneling is actually dominated by a single kind of pathway in both cases. This observation is consistent with the fact that almost all pathways run through Trp378 or Phe348. Note that $R_{coh}^{cl}$ is a classical coherence-like parameter in the sense that it is calculated from ε_*tot*_ provided by the semi-empirical pathway model. $R_{coh}^{cl}$ does not explicitly capture fine quantum effects like interferences among competing tunneling pathways running through the intervening medium (Prytkova et al., 2007). The pathway does not give access to *H*_*if*_, but to the decay ε_*tot*_ caused by the presence of an intervening medium (see the first equation). A crude estimation of the absolute coupling may still be obtained by setting $H_{if}^{contact}$ to 4.3 10^13^ Hz (0.177829 eV) (as in Prytkova et al., 2005), yielding an average value of 3.75 10^−6^ eV for $\langle H_{if}^{PM}\rangle$ from MD trajectories of the Fe^2+^/Fe^3+^ state.

### Probing the Putative O_2_-accessible Cavity and Tunneling Pathways

**Figure 7:**
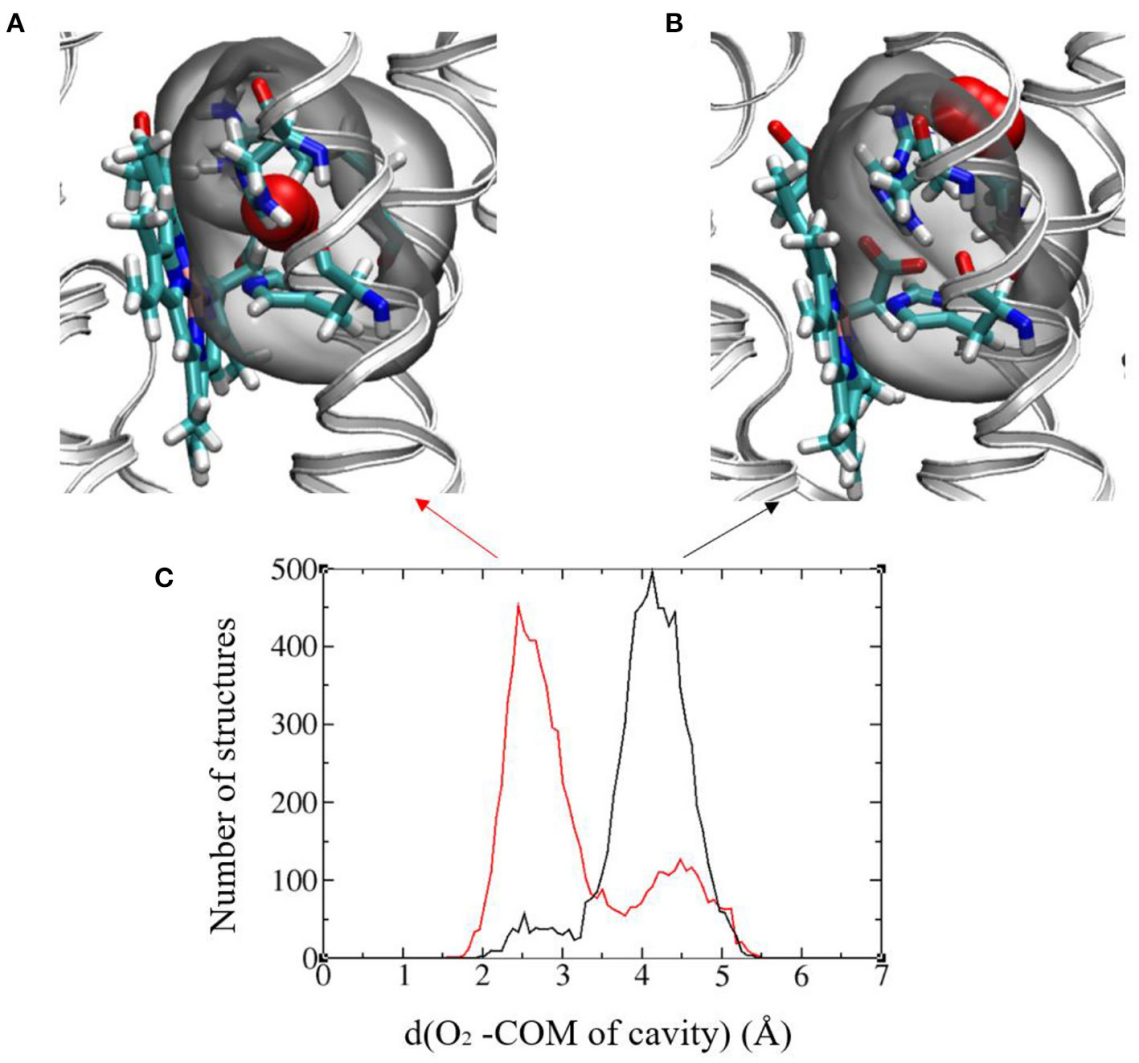
**(A)** and **(B)** Images showing the two favored O_2_ positions with respect to the binding cavity. The protein backbone is represented in gray ribbon and the cavity is highlighted in all-atom representation: Arg256, His313, and His317 and Heme2, O_2_ (in red). The cavity in gray bubble. **(C)** Histograms of the distance between O_2_ and the center of mass (COM) of the cavity in the initial (black) and final (red) redox states. Results from the 300ns-long MD runs.

The authors of Magnani et al. (2017) identified a putative cavity to bind the dioxygen molecule in the vicinity of Heme2 (right insert in Figure 2). In the simulation without O_2_, we saw that a water molecule occupied this cavity, that is shaped by residues Arg256, His313, and His317 and Heme2 (Figure 7). We replaced this water molecule with a dioxygen molecule to perform MD simulations with O_2_. First attempts to run simulations without constraint on O_2_ position lead to its escape from the protein matrix. This may be due to the fact that there is only one dioxygen molecule in the system and/or to the fact that the protein has not had enough time to accommodate to the O_2_ molecule. Since we are interested in the impact of O_2_ binding on the dynamics of the protein and on the ET parameters, we decided to constrain O_2_ to remain in the cavity. To this end we used Colvar options in NAMD. We defined a distance between the center of mass (COM) of the cavity and O_2_ [hereafter referred to as d(O_2_-COM)]. We added a boundary semi-harmonic potential restraint applying on this distance when it is more than 5 Å and with a force constant of 20 kcal/mol/Å^2^.

Figure 7C shows the histograms of this distance in the initial and final electronic states for the 300 ns-long MD simulations. A bimodal structure appears with two different positions of O_2_ around the cavity. The oscillations of O_2_ between these two positions occur at the timescale of few ns. The first position is O_2_ inserted inside the cavity with d(O_2_-COM) of around 2.5 Å (Figure 7A) and a distance between O_2_ and the Fe atom of Heme2 (d(O_2_-Fe)) around 5.5 Å. The other one corresponds to O_2_ lying on the edge of the cavity with d(O_2_-COM) of around 4.3 Å and d(O_2_-Fe) around 8.5 Å (Figure 7B). The relative proportion of occupancy of the two sites is reversed in the two electronic states. After the electron transfer (in red), O_2_ is more often close to Heme2 than in the initial state (black).

**Figure 8:**
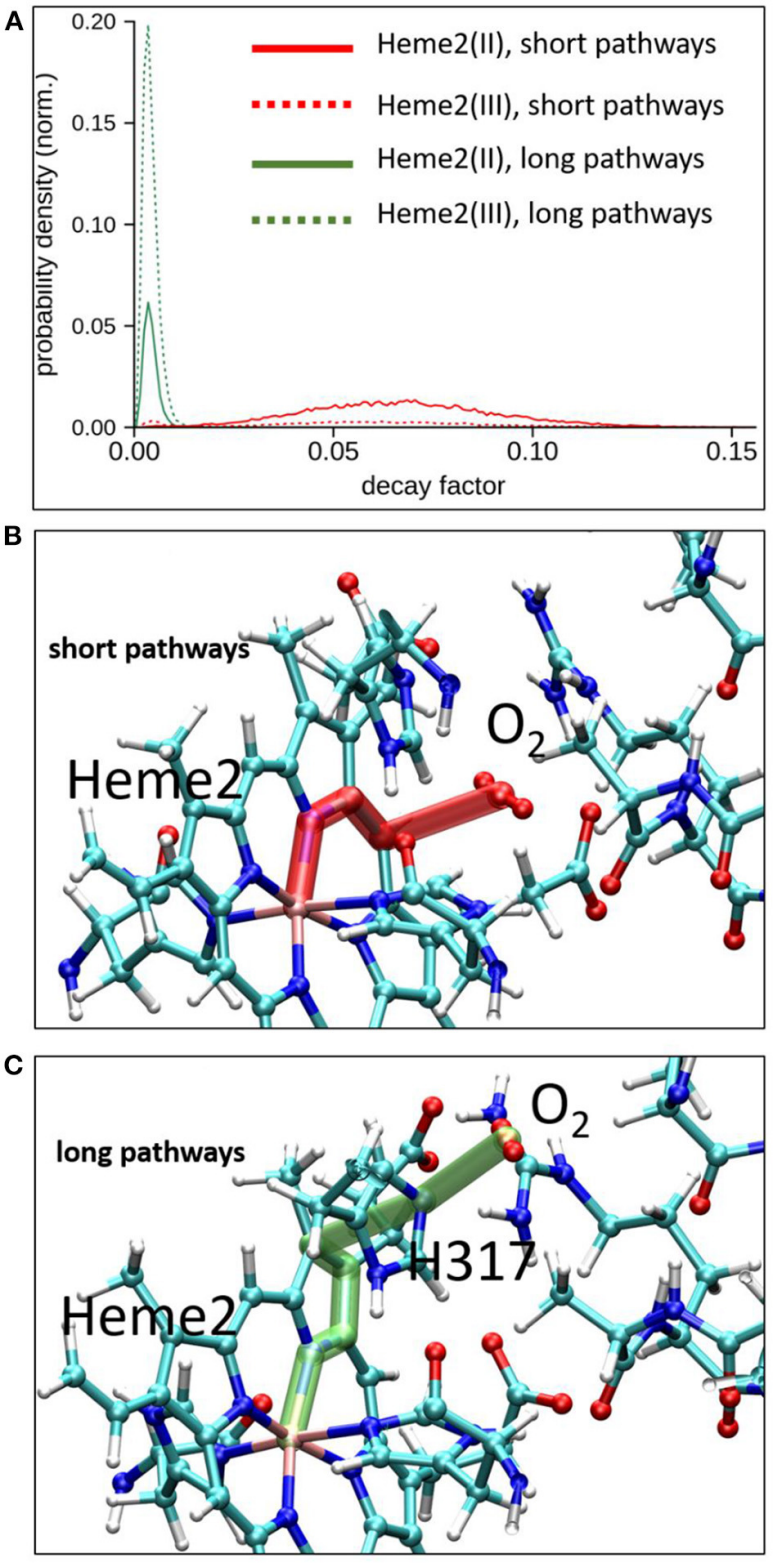
Electron tunneling pathway analysis between Heme2 and the dioxygen molecule. Top, normalized distributions of decay factors collected along the MD simulations in initial and final redox states of the inter-heme ET [(Heme2(III)) and Heme2(II) respectively]. Middle and bottom, representative snapshots of the two dominant pathways encountered during MD simulations.

To explore possible implications of the existence of these two connected cavities for electron tunneling pathways connecting Heme2 to O_2_, we have carried out pathway analyses as in the previous section, but enlarging the pruned system to encompass amino acid residues from the binding pocket. We have analyzed all the MD simulations (in the initial and final electronic states of the inter-heme ET). Two kinds of pathways are identified. The “short pathway” type implies a direct through-space jump from the porphyrin ring to O_2_. It is illustrated in Figure 8 (middle). The “long pathway” involves an intermediate passage through His317 before jumping to O_2_. The short pathways are associated to an average decay factor of 0.065 while the long pathways are associated to an average decay factor of 0.004, which is directly correlated to the respective lengths of the pathways. Interestingly, the short pathways are predominant when Heme2 is reduced, while the long pathways are dominant when Heme2 is oxidized. This could be a piece of the mechanism ensuring superoxide production catalysis. When Heme2 is reduced, thus ready to transfer an electron to a dioxygen molecule, the latter binds closer to the porphyrin ring, hence enabling very fast tunneling. The molecular origin of the dynamical control of the position shifting is not completely clear. From our simulations, it appears that the positively charged arginine Arg256 and histidine His317 play a role in this process. The conformations accessible to Arg256 in both redox states are similar. There is no frank residue repositioning upon Heme2 reduction. On the other hand, while Arg256 is almost exclusively hydrogen bonded to the propionate moieties in the ferrous state, it also often makes alternative hydrogen bonds to His317 in the ferric state (Supplementary Figure 8). In such situations, the cavity for O_2_ binding close to Heme2 is inaccessible. Further simulations will be needed to fully explain this behavior.

## Discussion and Conclusion

This study reports the first molecular simulations of a NOX system inserted into a lipid bilayer membrane. Starting from separate experimental structures of the DH and TM domains of NOX5, we have built a fused model of the full protein. This model is stable in MD simulations at the timescale of hundreds of nanoseconds. The interface between the two domains involves the same residues as in the very recent cryo-EM structures of the homologous protein DUOX1, however the DH domain has a different orientation in our model. We have focused on electron transfer between Heme1 and Heme2 within the TM domain, and on the impact of O_2_ on ET thermodynamics parameters. Dioxygen binding in the vicinity of Heme 2 appears to reduce the fluctuations of the energy difference between the two redox states involved in the ET, leading to more stable values of ET parameters. In both cases, O_2_ binding doesn't significantly alter the thermodynamics of inter-herme electron transfer. Negative values of the redox free energies on the order of few tenths of eV have been obtained, corresponding to spontaneous ET. Outer-sphere reorganization energies of about 1.1–1.3 eV (including contributions of the inner and outer-spheres) have been observed. Decomposition of these thermodynamics parameters over the molecular components of the system has provided additional insight. First, the DH has little impact on the inter-heme ET, suggesting that the relative orientation of the DH with respect to the cryo-EM structure is not a major concern for the present study of inter-heme ET. Second, we have highlighted the subtle balance existing between the protein matrix, the membrane, the water and the counter-ions. Our results should stimulate other studies aiming at investigating for instance the effect of membrane composition on inter-heme ET. Considering that outer-sphere reorganization energies have been calculated with a non-polarizable forcefield and are thus overestimated by *ca*. 30%, the values obtained fall within the range of values (0.7–1.0 eV) obtained recently for inter-heme ET within multi-hemic protein (Breuer et al., 2012). We have finally evaluated the tunneling coupling matrix element to fall within the μeV based on the tunneling pathway model (Beratan et al., 1991; Prytkova et al., 2005). We have been able to confirm the important role of Trp378 and Phe348 to sustain electron tunneling between the two hemes.

Dioxygen reduction by electron transfer is another essential part of NOX5 function that remains unclear though of prominent importance to understand the NOX machinery. It was suggested from Electron Paramagnetic Resonance and Raman spectroscopies (Yamaguchi et al., 1989; Hurst et al., 1991; Ueno et al., 1991; Fujii et al., 1999) that since Heme2 is hexacoordinated Heme2-to-O_2_ ET is likely to proceed via an outer-sphere mechanism. This hypothesis was corroborated by stopped-flow and rapid-scanning spectroscopy oxidation-reduction kinetics on porcine neutrophils (Isogai et al., 1995). The identification of a putative binding cavity in the 3D structure of NOX5 further supports this hypothesis (Magnani et al., 2017). In NOX5, the cavity is formed by Arg256, His313, and His317. We have investigated the localization of dioxygen near Heme2 by means of molecular dynamics simulations. Our simulations revealed that the cavity deduced from the X-ray structure is actually flexible, offering two possible binding sites, one being closer to the heme than the other, while the other is more accessible to solvent. The occupancy of these two sites depends on the redox states of the two hemes. When Heme2 is in a reduced state, the close position of O_2_ is largely favored. Importantly, this occupancy shift is coupled to the emergence of ten-times more efficient electron tunneling pathways than when O_2_ is positioned in the remote cavity. This 10-fold increase of tunneling matrix element would induce a 100-fold increase of ET rate form Heme2 to O_2_, therefore providing a powerful mean to catalyze superoxide production. We speculate that Arg256 is playing a central role in this process, since this positively charged residue necessarily rearranges when Heme2 is reduced. On the other hand, our MD simulations don't reveal any dramatic cavity shape modifications, but more subtle rearrangements. The stability of the hydrogen bond network among Arg256, His317, and the Heme2's propionate groups that shape the cavity appears to be sensitive to the charge present on Heme2. Further investigations will be needed to fully decipher this mechanism. Our study thus provides a possible mechanistic explanation to the experimental results reported in Magnani et al. (2017) indicating that the oxidation rate in R256S and H317R mutants were at least fivefold lower than observed for wild type. We now need to investigate the thermodynamics of this ET, including the possibility of spin-inversion on Heme2 cofactor as suggested by some authors (Doussière et al., 1996; Maturana et al., 2001), as well as the mechanism of primary binding of dioxygen into the remote cavity when Heme2 is oxidized.

This work is the first step toward a global investigation of the mechanism of superoxide production by the NOX machinery using molecular simulation. Many aspects deserve further analysis (computation of kinetic parameters, study of the electron transfers between FAD and Heme1, influence of lipid composition and/or mutation of key residues sustaining ETs in NOX…). The refinement of computational methods (such as the use of polarizable forcefields) will allow us to ascertain the conclusions drawn in this study, but the results obtained here open the path to a better understanding of NOX.

## Data Availability Statement

The datasets presented in this study can be found in online repositories. The names of the repository/repositories and accession number(s) can be found below: doi: 10.5281/zenodo.4424142; https://zenodo.org/record/4424142#.X_ciUyXjLeM/b.

## Author Contributions

MB, JH, and AL designed research. XW performed research. XW, JH, FC and AL carried out complementary analyses. All authors discussed and analyzed research data. All authors wrote the manuscript.

## Conflict of Interest

The authors declare that the research was conducted in the absence of any commercial or financial relationships that could be construed as a potential conflict of interest.
