# Supplementary material for: Mechanistic Insights on Heme-to-Heme Transmembrane Electron Transfer Within NADPH Oxydases From Atomistic Simulations
Source: Front Chem. 2021 May 4;9:650651. doi: 10.3389/fchem.2021.650651 (PMC8129163; doi:10.3389/fchem.2021.650651)
Supplement: Supplementary file 1 [file Data_Sheet_1.docx]

# Mechanistic Insights on the Heme-to-Heme Mechanistic Insights on Heme-to-Heme Transmembrane Electron Transfer within NADPH Oxydases from Atomistic Simulations

Xiaojing Wu^1,2,3^, Jérôme Hénin^1,2^, Laura Baciou^4^, Marc Baaden^1^, Fabien Cailliez^4^, Aurélien de la Lande^4^,

1: CNRS, Université de Paris, UPR 9080, Laboratoire de Biochimie Théorique, 13 rue Pierre et Marie Curie, F-75005, Paris, France

2 : Institut de Biologie Physico-Chimique-Fondation Edmond de Rotschild, PSL Research University, Paris, France

3, present address: Department of Physics and Astronomy, University College London, London WC1E 6BT, UK

4: Institut de Chimie Physique, Université Paris Saclay, CNRS (UMR 8000), 15 avenue Jean Perrin 91405, Orsay, France.

## Comparison between our model and recent cryo-EM structure of Duox-1

Figure S1 shows the comparison between our model of NOX5 (grey) and the recent cryo-EM structure of Duox1 (PDB code 7D3E). The two structures have been superimposed on their TM domain and two different views are presented.

| 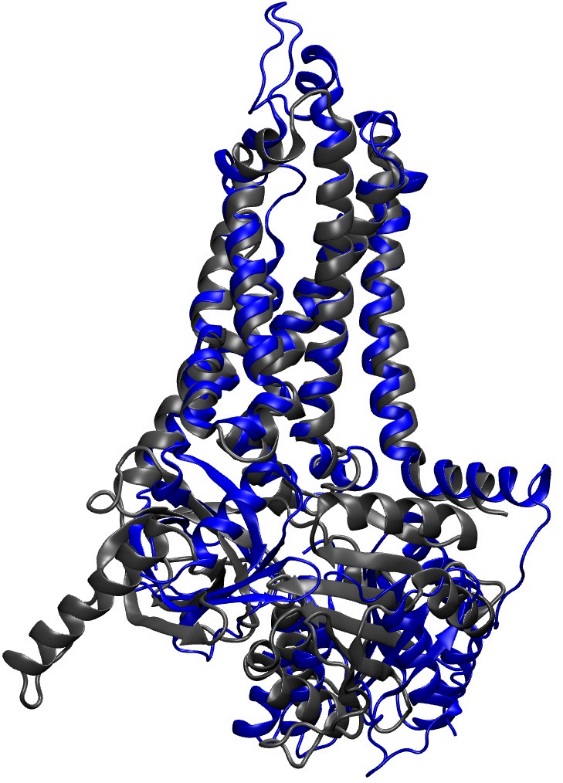 | 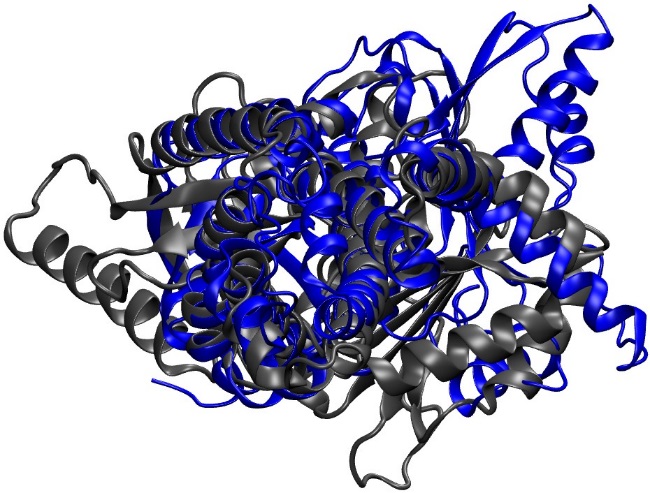 |
| --- | --- |
| (a) | (b) |

**Figure S1**: Two views of our NOX5 model structure (grey) and structure of Duox1 (blue), superimposed on their TM domain.

## Analysis of protein stability


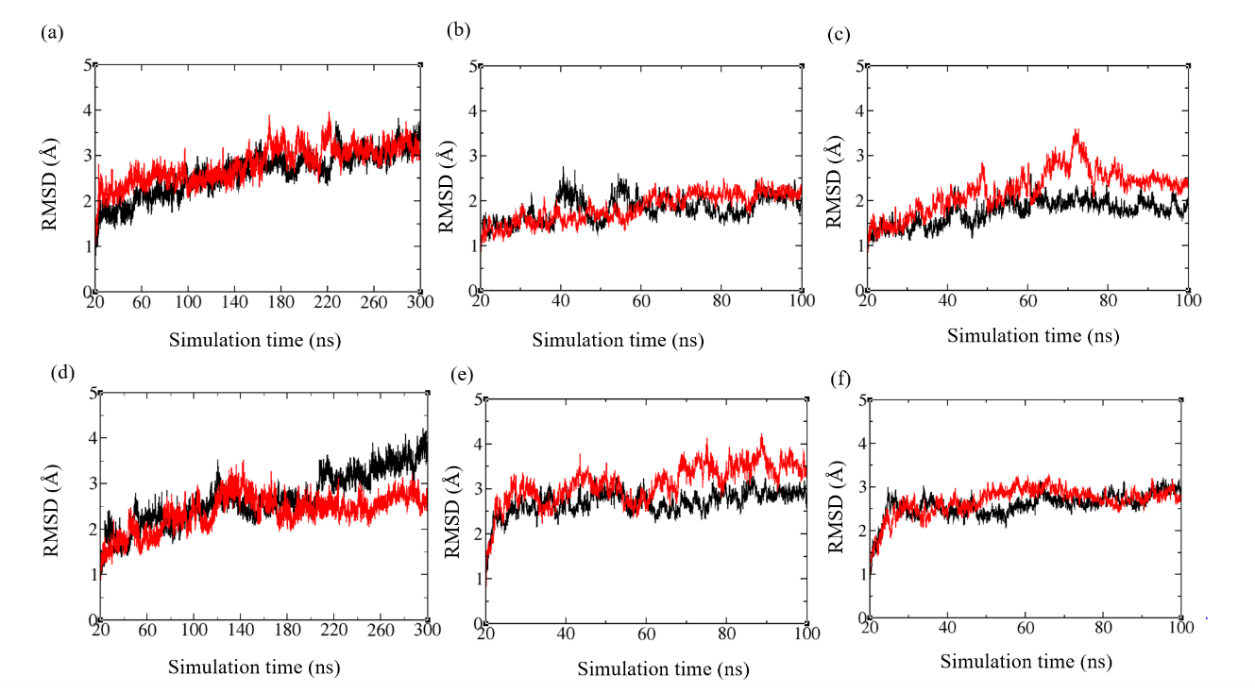


**Figure S2:** Evolution of RMSD of protein backbone in the initial (black) and final (red) redox states. (a), (b), (c): results of the three MD runs with O_2_. (d), (e), (f): results of the three MD runs without O_2_.


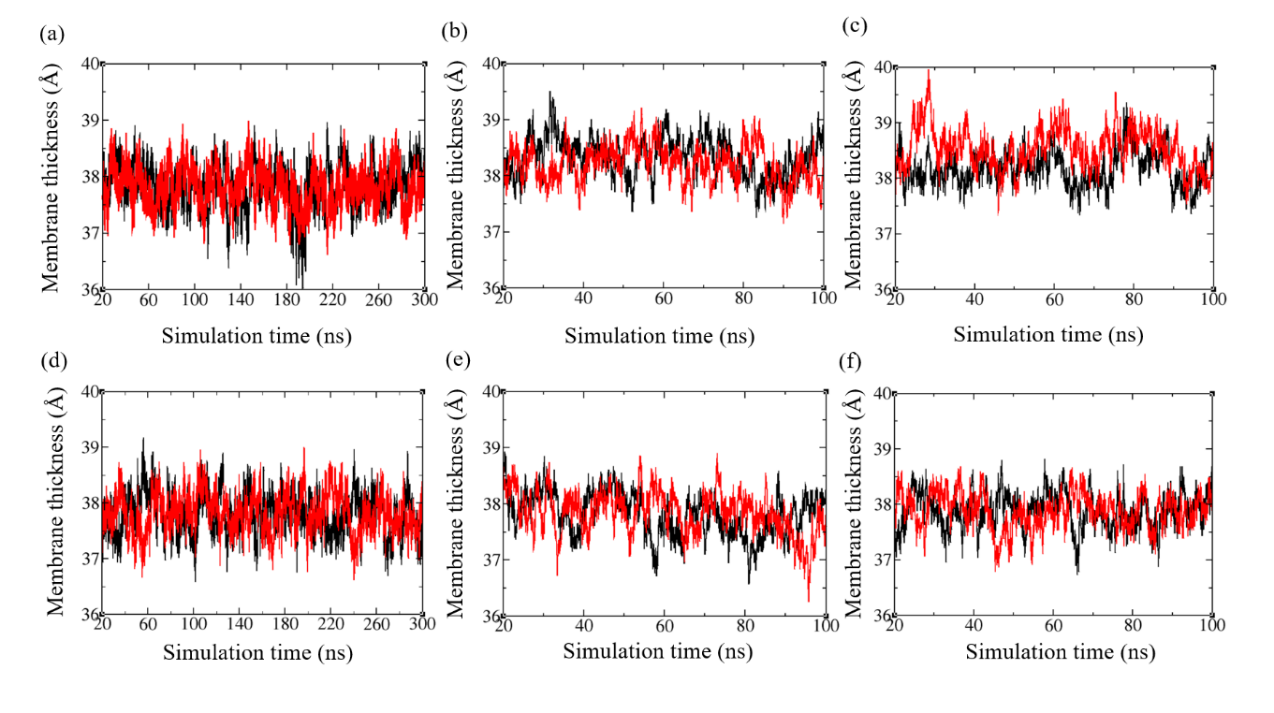


***Figure S3****: Fluctuation of membrane thickness in the initial (black) and final (red) redox states. (a), (b), (c): results of the three MD runs with O_2_. (d), (e), (f): results of the three MD runs without O_2_.*


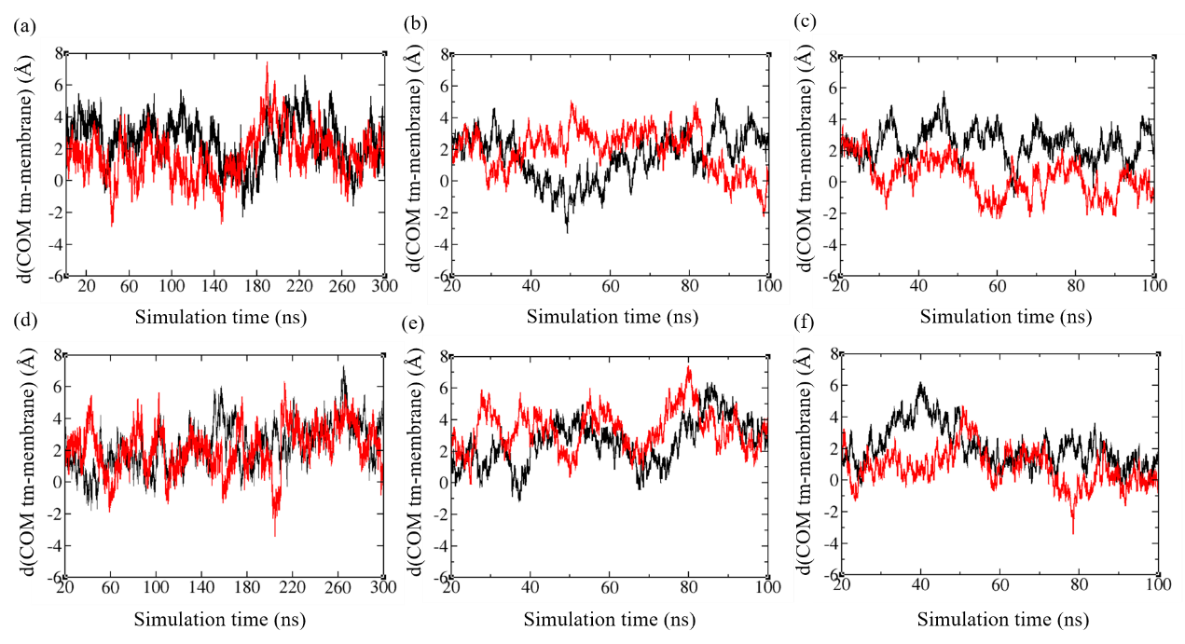


***Figure S4:*** *Fluctuation of the distance between the center of mass of the lipid bilayer and the center of mass of the TM domain in the initial (black) and final (red) redox states. Panels (a) to (c) are for MD simulations with O_2_, whereas panels (d) to (f) correspond to MD simulations without O_2_.*

## Thermodynamic parameters for electron transfer between Heme1 and Heme2

| **Dynamics** | $\Delta A^{\circ}$ | $\lambda^{St}$ | $\lambda_{i}^{var}$ | $\lambda_{f}^{var}$ | $\chi_{G}$ |
| --- | --- | --- | --- | --- | --- |
| **With O_2_** | | | | | |
| Set1 | -0.26±0.03 | 1.12±0.03 | 2.00±0.69 | 1.42±0.22 | 1.53 |
| Set 2 | -0.34±0.01 | 0.97±0.01 | 1.21±0.16 | 1.25±0.11 | 1.27 |
| Set 3 | -0.34±0.02 | 1.05±0.02 | 1.66±0.52 | 1.29±0.13 | 1.40 |
| Set 4 | -0.29±0.03 | 1.08±0.03 | 1.93±0.70 | 1.32±0.16 | 1.51 |
| Set 5 | -0.15±0.01 | 1.18±0.01 | 1.31±0.19 | 1.25±0.13 | 1.10 |
| Block Average | -0.28±0.07 | 1.08±0.07 | 1.62±0.32 | 1.31±0.03 | 1.36 |
| 300ns-long MD | -0.32±0.01 | 1.04±0.01 | 1.75±0.32 | 1.33±0.09 | 1.48 |
| **Without O_2_** | | | | | |
| Set 1 | -0.13±0.03 | 1.31±0.03 | 1.49±0.21 | 1.88±0.56 | 1.29 |
| Set 2 | -0.28±0.03 | 1.38±0.03 | 2.16±0.65 | 1.33±0.14 | 1.27 |
| Set 3 | -0.49±0.03 | 1.02±0.03 | 1.81±0.61 | 1.28±0.13 | 1.51 |
| Set 4 | -0.22±0.02 | 1.22±0.02 | 1.69±0.41 | 1.43±0.20 | 1.27 |
| Set 5 | -0.09±0.03 | 1.25±0.03 | 1.86±0.47 | 1.69±0.37 | 1.42 |
| Block Average | -0.24±0.14 | 1.24±0.24 | 1.80±0.22 | 1.52±0.03 | 1.35 |
| 300ns-long MD | -0.30±0.04 | 1.25±0.04 | 3.39±1.17 | 1.64±0.21 | 2.01 |

**Table S1:** ET reaction free energies and outer-sphere reorganization energies of the ET between hemes with O_2_ and without O_2_. All the energetic values are given in eV and the errors correspond to twice the uncertainty on the quantity. The last column corresponds to the ratio of reorganization free energies computed from variance of energy gap and LRA as defined in the main text.

## Decomposition of energy gap

To better understand the fluctuation of energy gap in Figure 3 (main text), we also calculated the energy gap of different part of the system: TM domain, DH domain, Environment (water, membrane, counter ions) and cofactors (FAD, heme, O2). The result of these decomposition of the case of with O2 is shown in Figure S5, while the result of without O2 is shown in Figure S6.

We begin by describing the results in presence of dioxygen (Figure S5). The data clearly shows that fluctuations of the energy gap are mainly from TM domain and Environment, while DH domain gives smaller contribution to the fluctuation. This is due to that the DH domain is far from two heme center. Cofactors also gives very small contribution to the fluctuations. Here the heme panel is the outer sphere interaction between two heme. The fluctuation of energy gap in both initial and final state are similar. However, after summing these two parts the final state (in green) is more stable than the initial state (in red). The bimodality of the initial redox state in Figure 3 is a result of a combination of fluctuation of TM domain and environment. For example, in set1, it is the decrease of energy gap of the environment that draw the whole energy gap down, while in the set3, it is the increase of the energy gap of TM domain that draw the energy gap up. In set4, it is the decrease of the energy gap of TM domain that draw the energy gap down. It is not just from the structure change of the TM part. We didn’t find RMSD or secondary structure change of protein that correlated to the fluctuation of the energy gap. We got similar result in the case of without O2 (Figure S6). The big change of the initial state in set 3 in Figure 3 (b) is due to change of the environment. However in set4, we the environment also has smaller energy gap but the energy gap of TM part increase. Thus the total energy gap in set 4 (Figure 3(b)) is similar to the others. There is a strong interaction between environment and TM domain.

We also decomposed the total energy gap and outer-sphere reorganization energy of the ET in Figure 4 (main text) into different part (Figure S7). Here we only plot the result of TM domain, DH domain, Environment which have stronger fluctuations of energy gap.The upper panel Figure S7(a,b,c) is result with O2, while the lower panel (d,f,e) is result without O2. The number of energy gap and reorganization energy are summarized in table S2. We see clear that in both case DH domain gives very small contribution to the free energy and reorganization energies. Although the orientation of TM and DH domain in our structure is a bit different than the recent cryo-EM structure of Duox1, this may have small influence to our ET result, since the contribution of DH domain is small.


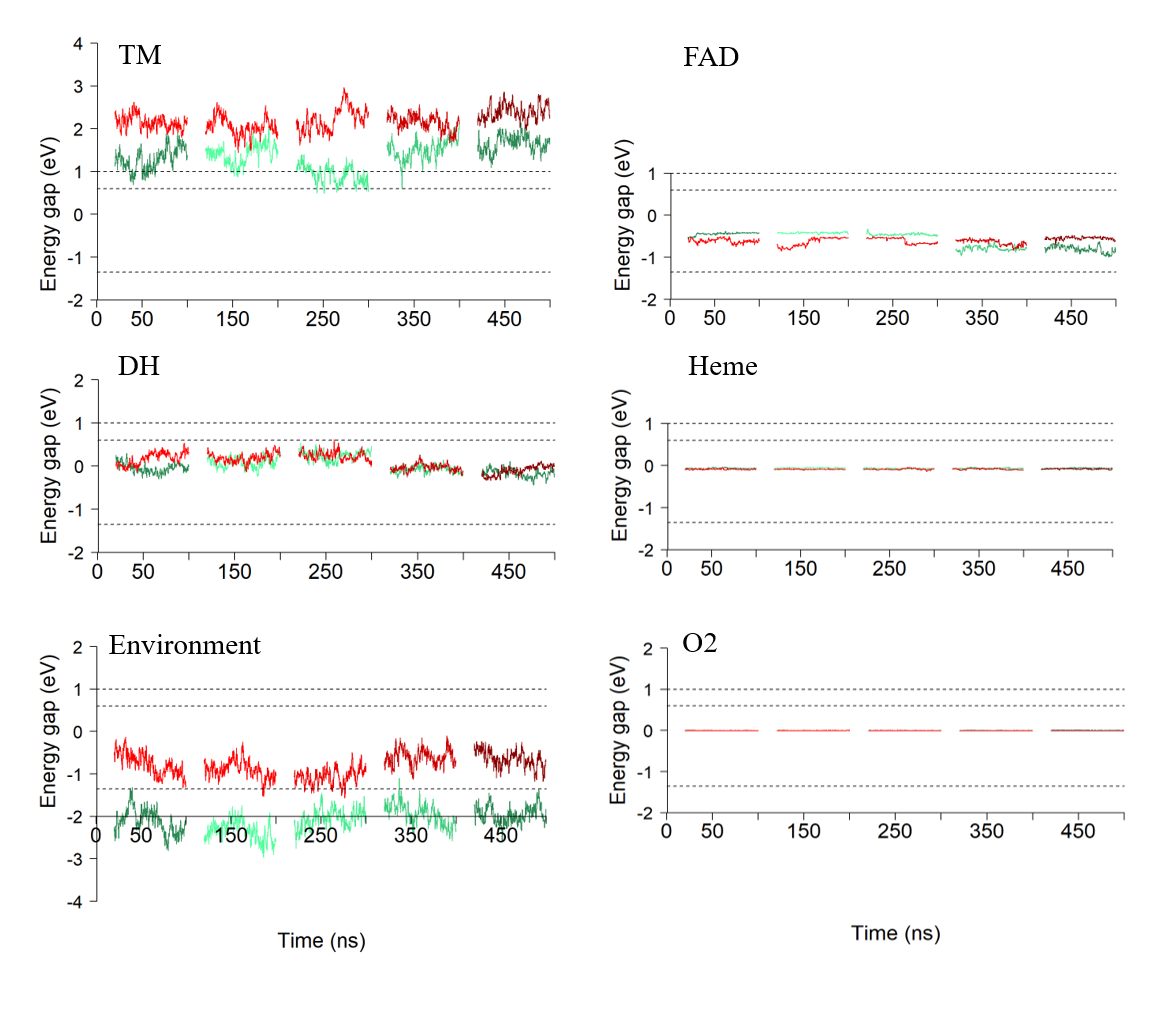


**Figure S5:** Evolution of the vertical energy gap $\Delta E$ ($E_{f}-E_{i}$) along MD simulations with O_2_ decomposition into different part. Each line section represents 80ns of MD simulation. The first three sections correspond to parts of the 300ns-long simulation (Set1 to Set3) while the 2 last sections come from the two independent 100ns-long replica simulations (Set4 and Set5). Computed energy gaps based on simulations in the initial (resp. final) redox state are shown in red (resp. green). Horizontal lines same as in Figure 3 are guides to the eye.


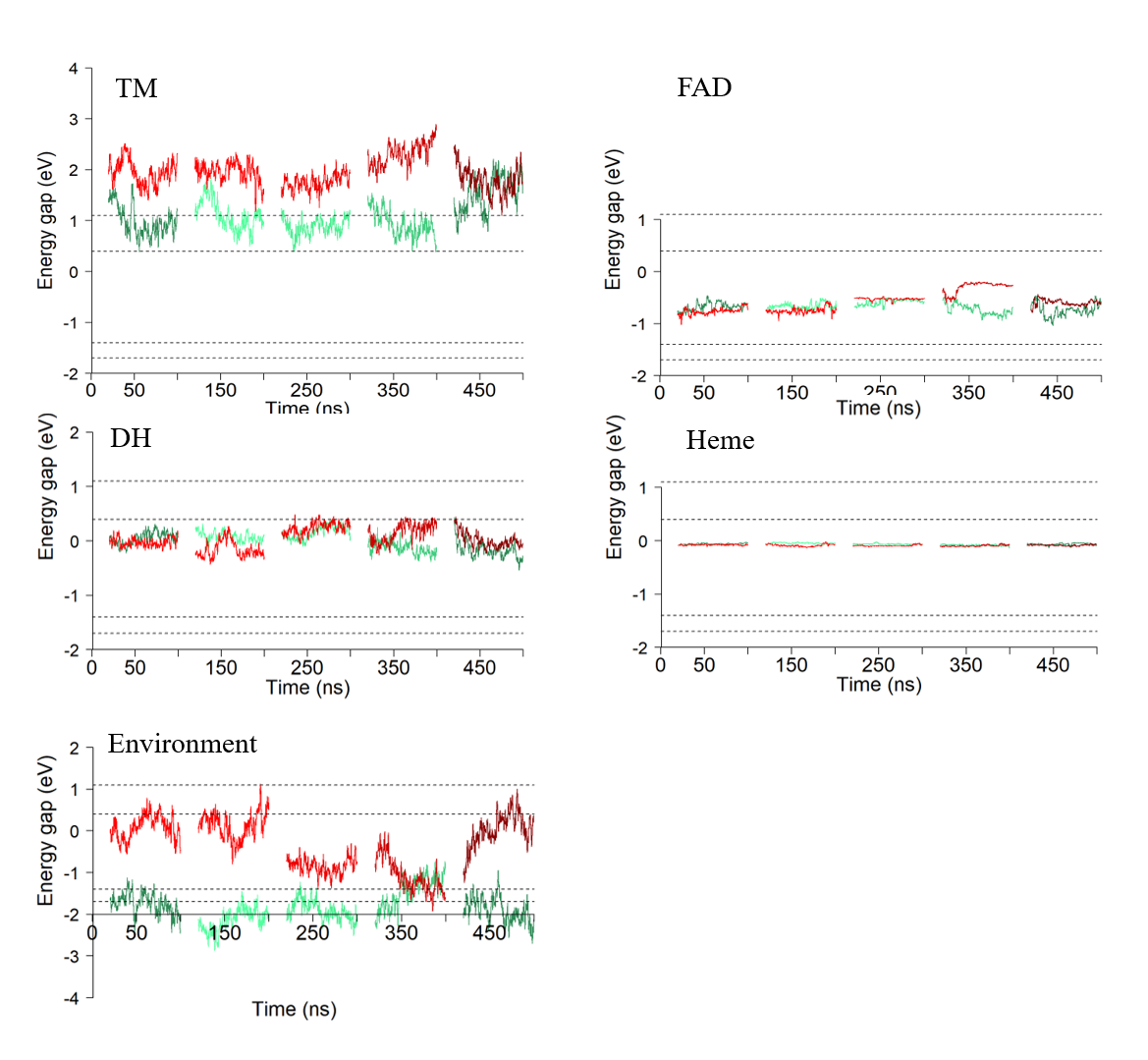


**Figure S6:** Evolution of the vertical energy gap $\Delta E$ ($E_{f}-E_{i}$) along MD simulations without O_2_ decomposition into different part. Each line section represents 80ns of MD simulation. The first three sections correspond to parts of the 300ns-long simulation (Set1 to Set3) while the 2 last sections come from the two independent 100ns-long replica simulations (Set4 and Set5). Computed energy gaps based on simulations in the initial (resp. final) redox state are shown in red (resp.green). Horizontal lines same as in Figure 3 are guides to the eye.


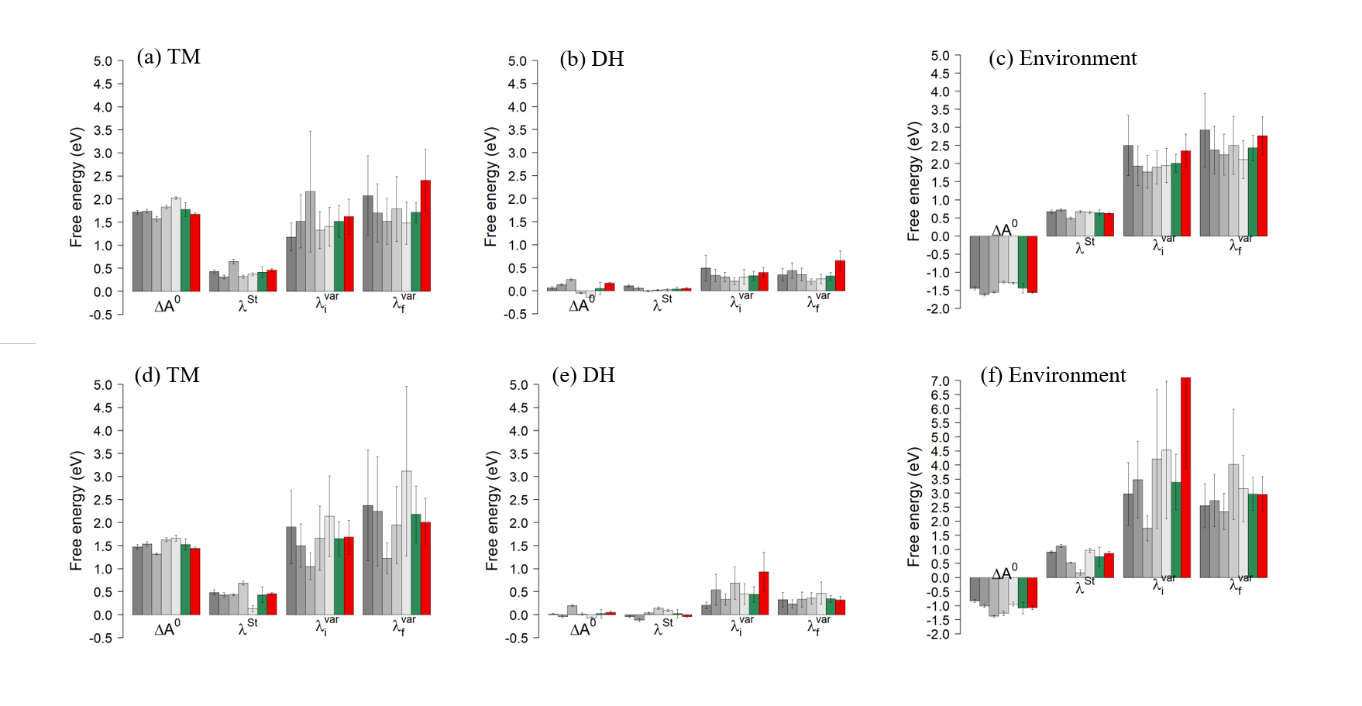


**Figure S7:** ET reaction free energies and outer-sphere reorganization energies of the ET between hemes with O2 (a,b,c) and without O_2_  (d,e,f) decomposed into TM domain, DH domain and environment. The grey bars represent values obtained for the five 100ns-long data sets. The green bars are the results of a block average using the data of these five data sets. The red bars correspond to values obtained using the full 300ns-long trajectory. Error bars correspond to twice the uncertainty.

|  |  | **Without O_2_** | | | **With O_2_** | | |
| --- | --- | --- | --- | --- | --- | --- | --- |
| **Part** | **Dynamics** | **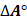** | **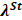** | Uncertainty | **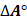** | **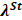** | Uncertainty |
| TM | TM |  |  |  |  |  |  |
|  | Set 1 | 1.47 | 0.48 | 0.03 | 1.72 | 0.43 | 0.02 |
|  | Set 2 | 1.54 | 0.43 | 0.02 | 1.74 | 0.32 | 0.02 |
|  | Set 3 | 1.32 | 0.44 | 0.01 | 1.58 | 0.65 | 0.03 |
|  | Set 4 | 1.63 | 0.69 | 0.02 | 1.83 | 0.32 | 0.02 |
|  | Set 5 | 1.67 | 0.13 | 0.03 | 2.03 | 0.37 | 0.01 |
|  | Block Average | 1.52 | 0.44 | 0.09 | 1.77 | 0.42 | 0.06 |
|  | 300ns-long MD | 1.44 | 0.45 | 0.01 | 1.68 | 0.46 | 0.01 |
| DH | Set 1 | 0.01 | -0.04 | 0.01 | 0.06 | 0.11 | 0.01 |
|  | Set 2 | -0.04 | -0.12 | 0.01 | 0.14 | 0.06 | 0.01 |
|  | Set 3 | 0.20 | 0.04 | 0.01 | 0.24 | 0.00 | 0.01 |
|  | Set 4 | 0.02 | 0.14 | 0.01 | -0.05 | 0.02 | 0.01 |
|  | Set 5 | -0.08 | 0.09 | 0.01 | -0.13 | 0.03 | 0.01 |
|  | Block Average | 0.02 | 0.02 | 0.05 | 0.07 | 0.04 | 0.02 |
|  | 300ns-long MD | 0.05 | -0.04 | 0.01 | 0.17 | 0.05 | 0.01 |
| FAD | Set 1 | -0.72 | -0.04 | 0.01 | -0.52 | -0.08 | 0.00 |
|  | Set 2 | -0.70 | -0.04 | 0.01 | -0.53 | -0.11 | 0.02 |
|  | Set 3 | -0.56 | 0.03 | 0.00 | -0.53 | -0.07 | 0.01 |
|  | Set 4 | -0.51 | 0.22 | 0.02 | -0.72 | 0.07 | 0.01 |
|  | Set 5 | -0.67 | 0.07 | 0.01 | -0.67 | 0.13 | 0.01 |
|  | Block Average | -0.63 | 0.04 | 0.05 | -0.59 | -0.02 | 0.05 |
|  | 300ns-long MD | -0.66 | -0.02 | 0.01 | -0.53 | -0.09 | 0.01 |
| Heme | Set 1 | -0.07 | -0.01 | 0.00 | -0.08 | -0.01 | 0.00 |
|  | Set 2 | -0.07 | -0.02 | 0.00 | -0.07 | -0.02 | 0.00 |
|  | Set 3 | -0.08 | -0.02 | 0.00 | -0.08 | -0.01 | 0.00 |
|  | Set 4 | -0.09 | -0.01 | 0.00 | -0.07 | -0.01 | 0.00 |
|  | Set 5 | -0.07 | -0.01 | 0.00 | -0.07 | -0.01 | 0.00 |
|  | Block Average | -0.07 | -0.01 | 0.00 | -0.07 | -0.01 | 0.00 |
|  | 300ns-long MD | -0.07 | -0.02 | 0.00 | -0.08 | -0.01 | 0.00 |
| Cl‑ | Set 1 | 0.61 | -0.07 | 0.04 | 0.49 | -0.07 | 0.03 |
|  | Set 2 | 0.39 | -0.01 | 0.04 | 0.45 | 0.03 | 0.04 |
|  | Set 3 | 0.38 | 0.01 | 0.03 | 0.41 | 0.05 | 0.04 |
|  | Set 4 | 0.32 | 0.24 | 0.04 | 0.55 | -0.04 | 0.04 |
|  | Set 5 | 0.57 | -0.09 | 0.04 | 0.57 | -0.09 | 0.03 |
|  | Block Average | 0.47 | 0.01 | 0.06 | 0.49 | -0.04 | 0.03 |
|  | 300ns-long MD | 0.49 | -0.02 | 0.02 | 0.45 | -0.03 | 0.02 |
| Na+ | Set 1 | -1.80 | 0.44 | 0.10 | -2.67 | 0.11 | 0.05 |
|  | Set 2 | -1.98 | 1.00 | 0.06 | -2.36 | 0.47 | 0.09 |
|  | Set 3 | -2.43 | -0.12 | 0.05 | -2.68 | -0.12 | 0.10 |
|  | Set 4 | -1.76 | -0.41 | 0.07 | -2.27 | 0.28 | 0.06 |
|  | Set 5 | -1.45 | 0.87 | 0.08 | -2.11 | -0.01 | 0.09 |
|  | Block Average | -1.89 | 0.37 | 0.28 | -2.45 | 0.12 | 0.10 |
|  | 300ns-long MD | -2.05 | 0.46 | 0.07 | -2.60 | 0.11 | 0.05 |
| Membrane | Set 1 | 0.39 | 0.21 | 0.08 | 0.90 | 0.19 | 0.06 |
|  | Set 2 | 1.04 | -0.18 | 0.09 | 0.59 | -0.06 | 0.13 |
|  | Set 3 | 0.83 | 0.16 | 0.07 | 0.96 | 0.14 | 0.07 |
|  | Set 4 | 0.60 | 0.16 | 0.08 | 0.56 | 0.01 | 0.08 |
|  | Set 5 | 0.29 | 0.02 | 0.06 | 0.30 | 0.23 | 0.05 |
|  | Block Average | 0.60 | 0.07 | 0.07 | 0.70 | 0.15 | 0.05 |
|  | 300ns-long MD | 0.69 | 0.06 | 0.06 | 0.85 | 0.17 | 0.06 |
| Water | Set 1 | -0.03 | 0.33 | 0.03 | -0.17 | 0.45 | 0.03 |
|  | Set 2 | -0.45 | 0.32 | 0.03 | -0.29 | 0.28 | 0.03 |
|  | Set 3 | -0.15 | 0.49 | 0.03 | -0.24 | 0.42 | 0.03 |
|  | Set 4 | -0.43 | 0.19 | 0.04 | -0.12 | 0.42 | 0.03 |
|  | Set 5 | -0.35 | 0.16 | 0.03 | -0.06 | 0.53 | 0.03 |
|  | Block Average | -0.27 | 0.30 | 0.06 | -0.20 | 0.41 | 0.04 |
|  | 300ns-long MD | -0.20 | 0.37 | 0.02 | -0.26 | 0.37 | 0.02 |

**Table S2:** ET reaction free energies and outer-sphere reorganization energies of the ET between hemes with O_2_ and without O_2_ decomposed into different part. Environment is the sum of contribution from water, membrane and counter ions. All energies values are given in eV. The uncertainty is the same for free energy and reorganization energy.

| 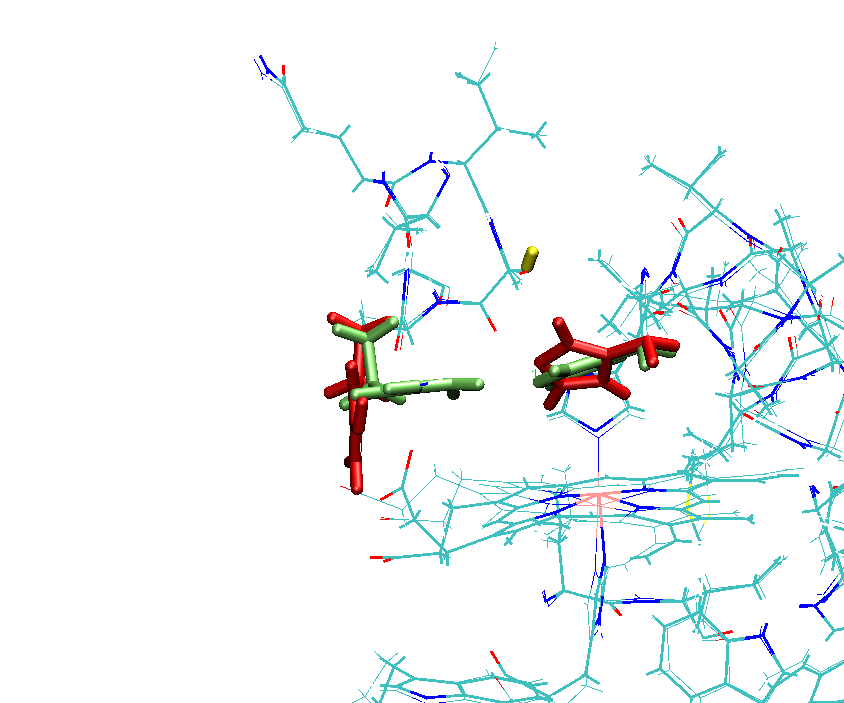 | 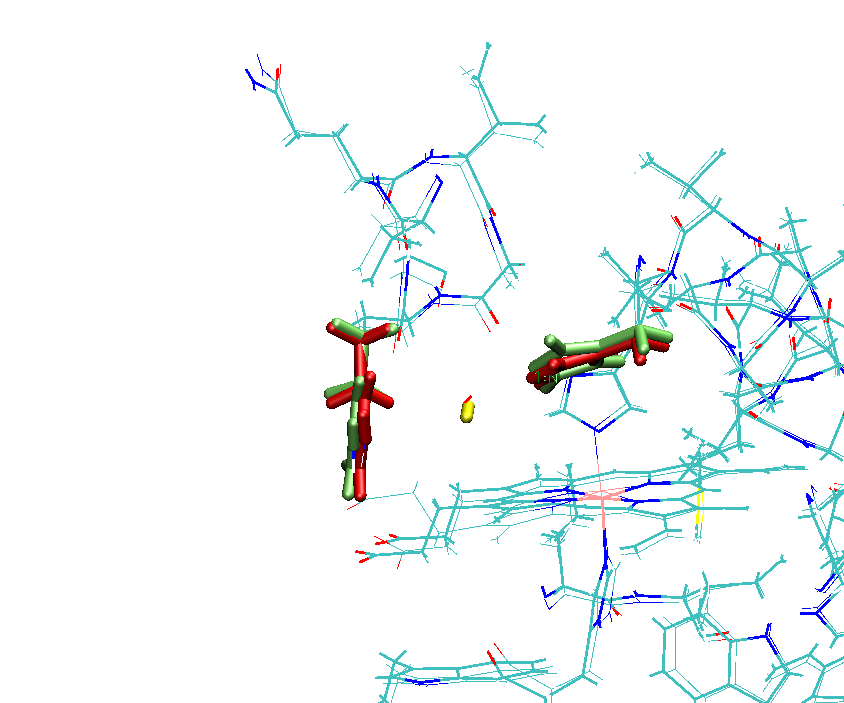 |
| --- | --- |

***Figure S8.*** *Two representative snapshots showing the conformations adopted by Arg256 and His317 in the simulations of reduced Heme2 (red) or oxidized Heme2 (green). Dioxygen is shown in yellow. Left, Arg256 either makes hydrogen bonds with the Heme2 propionate groups (red), of make an hydrogen bond with His317 (green). Right, Arg256 only makes hydrogen bonds with the propionates. O_2_ is present within the cavity.*

## Charge parameters for cofactors

| HEME and 2 HIS ligands | | | | |
| --- | --- | --- | --- | --- |
| Resname | Atom | Type | Ferrous | Ferric |
| HEME | FE | FE | 1.6873 | 1.7946 |
| HEME | NA | NPH | -0.6092 | -0.6250 |
| HEME | NB | NPH | -0.6062 | -0.6202 |
| HEME | NC | NPH | -0.6280 | -0.6444 |
| HEME | ND | NPH | -0.6341 | -0.6500 |
| HEME | C1A | CPA | 0.3146 | 0.3184 |
| HEME | C2A | CPB | -0.0872 | -0.0439 |
| HEME | C3A | CPB | -0.0117 | 0.0084 |
| HEME | C4A | CPA | 0.2937 | 0.3122 |
| HEME | C1B | CPA | 0.2845 | 0.3049 |
| HEME | C2B | CPB | 0.0310 | 0.0418 |
| HEME | C3B | CPB | -0.1380 | -0.0902 |
| HEME | C4B | CPA | 0.3283 | 0.3284 |
| HEME | C1C | CPA | 0.2920 | 0.2981 |
| HEME | C2C | CPB | 0.0436 | 0.0703 |
| HEME | C3C | CPB | -0.1547 | -0.1232 |
| HEME | C4C | CPA | 0.3232 | 0.3361 |
| HEME | C1D | CPA | 0.2920 | 0.3090 |
| HEME | C2D | CPB | -0.0040 | 0.0179 |
| HEME | C3D | CPB | -0.0945 | -0.0544 |
| HEME | C4D | CPA | 0.3198 | 0.3242 |
| HEME | CHA | CPM | -0.4064 | -0.3912 |
| HEME | HA | HA | 0.1458 | 0.1626 |
| HEME | CHB | CPM | -0.4036 | -0.3975 |
| HEME | HB | HA | 0.1427 | 0.1609 |
| HEME | CHC | CPM | -0.3891 | -0.3741 |
| HEME | HC | HA | 0.1401 | 0.1568 |
| HEME | CHD | CPM | -0.4277 | -0.4201 |
| HEME | HD | HA | 0.1470 | 0.1643 |
| HEME | CMA | CT3 | -0.4525 | -0.4751 |
| HEME | HMA1 | HA3 | 0.1438 | 0.1624 |
| HEME | HMA2 | HA3 | 0.1388 | 0.1576 |
| HEME | HMA3 | HA3 | 0.1376 | 0.1491 |
| HEME | CAA | CT2 | -0.2352 | -0.2118 |
| HEME | HAA1 | HA2 | 0.1115 | 0.1208 |
| HEME | HAA2 | HA2 | 0.1212 | 0.1374 |
| HEME | CBA | CT2 | -0.2800 | -0.2800 |
| HEME | HBA1 | HA2 | 0.1621 | 0.1641 |
| HEME | HBA2 | HA2 | 0.1598 | 0.1560 |
| HEME | CGA | CC | 0.6200 | 0.6200 |
| HEME | O1A | OC | -0.7600 | -0.7600 |
| HEME | O2A | OC | -0.7600 | -0.7600 |
| HEME | CMB | CT3 | -0.4664 | -0.4870 |
| HEME | HMB1 | HA3 | 0.1386 | 0.1548 |
| HEME | HMB2 | HA3 | 0.1352 | 0.1480 |
| HEME | HMB3 | HA3 | 0.1521 | 0.1682 |
| HEME | CAB | CE1 | -0.0362 | -0.0725 |
| HEME | HAB | HE1 | 0.1013 | 0.1160 |
| HEME | CBB | CE2 | -0.3583 | -0.2999 |
| HEME | HBB1 | HE2 | 0.1300 | 0.1454 |
| HEME | HBB2 | HE2 | 0.1419 | 0.1484 |
| HEME | CMC | CT3 | -0.4672 | -0.4929 |
| HEME | HMC1 | HA3 | 0.1430 | 0.1590 |
| HEME | HMC2 | HA3 | 0.1379 | 0.1507 |
| HEME | HMC3 | HA3 | 0.1500 | 0.1688 |
| HEME | CAC | CE1 | -0.0355 | -0.0667 |
| HEME | HAC | HE1 | 0.0977 | 0.1119 |
| HEME | CBC | CE2 | -0.3642 | -0.3098 |
| HEME | HBC1 | HE2 | 0.1286 | 0.1452 |
| HEME | HBC2 | HE2 | 0.1399 | 0.1477 |
| HEME | CMD | CT3 | -0.4719 | -0.4950 |
| HEME | HMD1 | HA3 | 0.1398 | 0.1538 |
| HEME | HMD2 | HA3 | 0.1370 | 0.1515 |
| HEME | HMD3 | HA3 | 0.1540 | 0.1723 |
| HEME | CAD | CT2 | -0.2415 | -0.2146 |
| HEME | HAD1 | HA2 | 0.1263 | 0.1425 |
| HEME | HAD2 | HA2 | 0.1153 | 0.1244 |
| HEME | CBD | CT2 | -0.2800 | -0.2800 |
| HEME | HBD1 | HA2 | 0.1570 | 0.1691 |
| HEME | HBD2 | HA2 | 0.1613 | 0.1602 |
| HEME | CGD | CC | 0.6200 | 0.6200 |
| HEME | O1D | OC | -0.7600 | -0.7600 |
| HEME | O2D | OC | -0.7600 | -0.7600 |
| HSD | CB | CT2 | -0.5329 | -0.5551 |
| HSD | HB1 | HA2 | 0.1719 | 0.1832 |
| HSD | HB2 | HA2 | 0.1503 | 0.1680 |
| HSD | ND1 | NR1 | -0.4409 | -0.4294 |
| HSD | HD1 | H | 0.3530 | 0.3700 |
| HSD | CG | CPH1 | 0.2740 | 0.3044 |
| HSD | CE1 | CPH2 | -0.0843 | -0.0836 |
| HSD | HE1 | HR1 | 0.1294 | 0.1358 |
| HSD | NE2 | NR2 | -0.5227 | -0.5315 |
| HSD | CD2 | CPH1 | 0.2721 | 0.2920 |
| HSD | HD2 | HR3 | 0.0968 | 0.1057 |
| HSD | CB | CT2 | -0.5332 | -0.5554 |
| HSD | HB1 | HA2 | 0.1498 | 0.1679 |
| HSD | HB2 | HA2 | 0.1721 | 0.1835 |
| HSD | ND1 | NR1 | -0.4412 | -0.4299 |
| HSD | HD1 | H | 0.3528 | 0.3702 |
| HSD | CG | CPH1 | 0.2752 | 0.3065 |
| HSD | CE1 | CPH2 | 0.2711 | 0.2911 |
| HSD | HE1 | HR1 | 0.0979 | 0.1083 |
| HSD | NE2 | NR2 | -0.5216 | -0.5305 |
| HSD | CD2 | CPH1 | -0.0852 | -0.0854 |
| HSD | HD2 | HR3 | 0.1316 | 0.1395 |

| FADH• | | | |
| --- | --- | --- | --- |
| Resname | Atom | Type | charge |
| FLAR | C7 | CAP | 0.200 |
| FLAR | C7M | CT3 | -0.566 |
| FLAR | H71 | HA | 0.187 |
| FLAR | H72 | HA | 0.163 |
| FLAR | H73 | HA | 0.192 |
| FLAR | C8 | CAP | 0.200 |
| FLAR | C8M | CT3 | -0.564 |
| FLAR | H81 | HA | 0.173 |
| FLAR | H82 | HA | 0.176 |
| FLAR | H83 | HA | 0.180 |
| FLAR | C6 | CAP | -0.361 |
| FLAR | H6 | HP | 0.179 |
| FLAR | C9 | CAP | -0.347 |
| FLAR | H9 | HP | 0.166 |
| FLAR | C5A | CAP | 0.252 |
| FLAR | N5 | NR2 | -0.347 |
| FLAR | H5 | H | 0.399 |
| FLAR | C4A | CAP | -0.225 |
| FLAR | C4 | CAP | 0.737 |
| FLAR | O4 | O | -0.708 |
| FLAR | N3 | NR2 | -0.826 |
| FLAR | H3 | H | 0.449 |
| FLAR | C2 | CAP | 1.005 |
| FLAR | O2 | O | -0.722 |
| FLAR | N1 | NR2 | -0.725 |
| FLAR | C10 | CAP | 0.465 |
| FLAR | N10 | NR2 | -0.053 |
| FLAR | C9A | CAP | 0.165 |
| FLAR | CR1 | CN7 | -0.258 |
| FLAR | HR11 | HN7 | 0.120 |
| FLAR | HR12 | HN7 | 0.147 |
| FLAR | CR2 | CN7 | 0.205 |
| FLAR | HR21 | HN7 | 0.071 |
| FLAR | OR2 | ON5 | -0.629 |
| FLAR | HR22 | HN5 | 0.477 |
| FLAR | CR3 | CN7 | 0.110 |
| FLAR | HR31 | HN7 | 0.068 |
| FLAR | OR3 | ON5 | -0.638 |
| FLAR | HR32 | HN5 | 0.470 |
| FLAR | CR4 | CN7 | 0.220 |
| FLAR | HR41 | HN7 | 0.078 |
| FLAR | OR4 | ON5 | -0.586 |
| FLAR | HR42 | HN5 | 0.409 |
| FLAR | CR5 | CN7 | -0.354 |
| FLAR | HR51 | HN7 | 0.200 |
| FLAR | HR52 | HN7 | 0.206 |
| ADP | C4' | CN7 | 0.160 |
| ADP | H4' | HN7 | 0.090 |
| ADP | O4' | ON6B | -0.500 |
| ADP | C1' | CN7B | 0.160 |
| ADP | H1' | HN7 | 0.090 |
| ADP | C5 | CN5 | 0.280 |
| ADP | N7 | NN4 | -0.710 |
| ADP | C8 | CN4 | 0.340 |
| ADP | H8 | HN3 | 0.120 |
| ADP | N9 | NN2 | -0.050 |
| ADP | N1 | NN3A | -0.740 |
| ADP | C2 | CN4 | 0.500 |
| ADP | H2 | HN3 | 0.130 |
| ADP | N3 | NN3A | -0.750 |
| ADP | C4 | CN5 | 0.430 |
| ADP | C6 | CN2 | 0.460 |
| ADP | N6 | NN1 | -0.770 |
| ADP | H61 | HN1 | 0.380 |
| ADP | H62 | HN1 | 0.380 |
| ADP | C2' | CN7B | 0.140 |
| ADP | H2'' | HN7 | 0.090 |
| ADP | O2' | ON5 | -0.660 |
| ADP | H2' | HN5 | 0.430 |
| ADP | C3' | CN7 | 0.140 |
| ADP | H3' | HN7 | 0.090 |
| ADP | O3' | ON5 | -0.660 |
| ADP | H3T | HN5 | 0.430 |
| ADP | C5' | CN8B | -0.080 |
| ADP | H5' | HN8 | 0.090 |
| ADP | H5'' | HN8 | 0.090 |
| ADP | O5' | ON2 | -0.620 |
| ADP | PA | P | 1.500 |
| ADP | O1A | ON3 | -0.820 |
| ADP | O2A | ON3 | -0.820 |
| ADP | O3A | ON2 | -0.740 |
| ADP | PB | P | 1.500 |
| ADP | O1B | ON3 | -0.820 |
| ADP | O2B | ON3 | -0.820 |
| ADP | O3B | ON2 | -0.620 |

| O2 | | | |
| --- | --- | --- | --- |
| Resname | Atom | Type | charge |
| O2Q | O1 | OM | -0.226 |
| O3Q | OG | OG | 0.452 |
| O4Q | O2 | OM | -0.226 |

**Density Functional Theory calculations for inner-sphere contributions**

Density Functional Theory (DFT) calculations have been carried out with the deMon2k software^1^ to evaluate the inner-sphere reorganization energy. Heme complexes have been optimized within the Kohn-Sham Auxiliary DFT framework^2^. To conduct geometry optimizations, we used the PBE exchange-correlation energy functional in combination with the DZVP-GGA basis set^3^. The variationally fitted densities have been expanded over the automatically generated GEN-A2 basis for H and GEN-A2* for Fe, C, N and O atoms. The XC contributions were evaluated with numerical grid of very high accuracy (10^-10^ Ha). Single energy calculations have been carried out with a TZVP basis set and GEN-A2* on all atoms.

**REFERENCES**

(1) Köster, A. M.; Geudtner, G.; Alvarez-Ibarra, A.; Calaminici, P.; Casida, M. E.; Carmona-Espindola, J.; Dominguez, V.; Flores-Moreno, R.; Gamboa, G. U.; Goursot, A.; Heine, T.; Ipatov, A.; de la Lande, A.; Janetzko, F.; del Campo, J.-M.; Mejia-Rodriguez, D.; Reveles, J.; Vasquez-Perez, J.; Vela, A.; Zuniga-Gutierrez, B.; Salahub, D. R. *DeMon2k Version 5*; Mexico City, 2018.

(2) Calaminici, P.; Alvarez-Ibarra, A.; Cruz-Olvera, D.; Domı́nguez-Soria, V.-D.; Flores-Moreno, R.; Gamboa, G. U.; Geudtner, G.; Goursot, A.; Mejı́a-Rodrı́guez, D.; Salahub, D. R.; Zuniga-Gutierrez, B.; ​Köster, A. Auxiliary Density Functional Theory: From Molecules to Nanostructures. In *Handbook of Computational Chemistry*; Leszczynski, J., Ed.; Springer Netherlands: Dordrecht, 2016; pp 1–67.

(3) Calaminici, P.; Janetzko, F.; Köster, A. M.; Mejia-Olvera, R.; Zuniga-Gutierrez, B. Density Functional Theory Optimized Basis Sets for Gradient Corrected Functionals: 3d Transition Metal Systems. *J. Chem. Phys.* **2007**, *126* (4), 044108. https://doi.org/doi:http://dx.doi.org/10.1063/1.2431643.
